# A four-part working bibliography of neuroethics: part 2 – neuroscientific studies of morality and ethics

**DOI:** 10.1186/s13010-015-0022-0

**Published:** 2015-02-15

**Authors:** Martina Darragh, Liana Buniak, James Giordano

**Affiliations:** Bioethics Research Library, Kennedy Institute of Ethics, Georgetown University, Washington, DC 20057 USA; Neuroethics Studies Program, Edmund D. Pellegrino Center for Clinical Bioethics, Georgetown University Medical Center, Washington, DC 20057 USA; Department of Neurology, Georgetown University Medical Center, Washington, DC 20057 USA; Human Science Center, Ludwig-Maximilians Universität, München, Germany

**Keywords:** Neuroethics, Neuroscience, Morality, Moral Psychology, Ethics, Bibliography

## Abstract

**Background:**

Moral philosophy and psychology have sought to define the nature of right and wrong, and good and evil. The industrial turn of the twentieth century fostered increasingly technological approaches that conjoined philosophy to psychology, and psychology to the natural sciences. Thus, moral philosophy and psychology became ever more vested to investigations of the anatomic structures and physiologic processes involved in cognition, emotion and behavior - ultimately falling under the rubric of the neurosciences. Since 2002, neuroscientific studies of moral thought, emotions and behaviors have become known as – and a part of – the relatively new discipline of neuroethics. Herein we present Part 2 of a bibliography of neuroethics from 2002–2013 addressing the “neuroscience of ethics” – studies of putative neural substrates and mechanisms involved in cognitive, emotional and behavioral processes of morality and ethics.

**Methods:**

A systematic survey of the neuroethics literature was undertaken. Bibliographic searches were performed by accessing 11 databases, 8 literature depositories, and 4 individual journal searches, and employed indexing language for National Library of Medicine (NLM) Medical Subject Heading databases. All bibliographic searches were conducted using the *RefWorks* citation management program.

**Results:**

This bibliography lists 397 articles, 65 books, and 52 book chapters that present (1) empirical/experimental studies, overviews, and reviews of neural substrates and mechanisms involved in morality and ethics, and/or (2) reflections upon such studies and their implications. These works present resources offering iterative descriptions, definitions and criticisms of neural processes involved in moral cognition and behaviors, and also provide a historical view of this field, and insights to its developing canon.

## Introduction and background

Throughout much of recorded history, humans have sought to define the nature of right and wrong, and good and evil. Since antiquity, such questions have been the focus of moral philosophy. However, empirical and experimental movements of the late nineteenth century drew scientific attention to philosophical questions, and the queries of moral philosophy became the focus of the then nascent discipline of psychology. The industrial turn of the twentieth century fostered increasingly technological approaches that conjoined psychology to the natural sciences. Philosophical speculation, and psychological observation and experimentation became ever more rooted in, and vested to investigations of the anatomic structures and physiologic processes involved in cognition, emotion and behavior. Thus, studies of moral philosophy and moral psychology became the province of brain research, ultimately falling under the rubric of the neurosciences, which became firmly established as a titular field in the middle-to-late 1970s [1]. Important contributory literature from the 1960s through early 2000s is provided below.

### Important contributory literature from the 1960s through early 2000s

#### Journal Articles

- Allison T: **Neuroscience and morality.***Neuroscientist* 2001, **7**(5):360-364.
- Callahan D: **Ethical responsibility in science in the face of uncertain consequences.***Ann N Y Acad Sci* 1976, **265**:1-12.
- Changeux JP: [**Reflections of a neurobiologist on the origin of ethics**.] *CR Seances Soc Biol Fil* 1998, **192**(6):1041-1049.
- Churchland PS: **The significance of neuroscience for philosophy.***Trends Neurosci* 1988, **11**(7): 304-307
- Damasio H, Grabowski T, Frank R, Galaburda AM, Damasio AR: **The return of Phineas Gage: clues about the brain from the skull of a famous patient.***Science* 1994, **264**(5162): 1102-1105.
- Dolan RJ: **On the neurology of morals**. *Nat Neurosci* 1999, **2**(11):927-929.
- Eslinger PJ, Damasio AR: **Severe disturbance of higher cognition after bilateral frontal lobe ablation: patient EVR.***Neurology* 1985, **35**(12):1731-1741.
- Greene JD, Sommerville RB, Nystrom LE, Darley JM, Cohen JD: **An fMRI investigation of emotional engagement in moral judgment.***Science* 2001, **293**(5537): 2105-2108.
- Helmuth L: **Cognitive neuroscience. Moral reasoning relies on emotion.***Science* 2001, **293**(5537):1971-1972.
- Laplane D. [**Epistemological remarks on the question of cerebral organization.**] *Rev Neurol* 1994, **150**(8-9):555-563.
- Medinnus GR: **Behavioral and cognitive measures of conscience development.** *J Genet Psychol* 1966, **109**(1):147-150.
- Scoville WB, Milner B: **Loss of recent memory after bilateral hippocampal lesions.***J Neurol Neurosurg Psychiat* 1957, **20**:11-21.
- Strawson G: **The impossibility of moral responsibility.***Philos Stud* 1994, **75**(1/2): 5-24.

#### Books

- Bratman M: *Intention, Plans, and Practical Reason*. Cambridge, Mass: Harvard University Press 1987.
- Brentano F: *Psychology from an Empirical Standpoint*. New York: Humanities Press 1973 (1874).
- Churchland PM: *The Engine of Reason, the Seat of the Soul: A Philosophical Journey into the Brain.* Cambridge, Mass: MIT Press 1995.
- Clark A: *Being There: Putting Brain, Body and World Together Again*. Cambridge, Mass.: MIT Press 1997.
- Crick F: *The Astonishing Hypothesis: The Scientific Search for the Soul.* New York: Touchstone 1994.
- Damasio AR: *Descartes' Error: Emotion, Reason and the Human Brain*. New York: Putnam 1994.
- Damasio AR: *The Feeling of What Happens: Body and Emotion in the Making of Consciousness*. New York: Harcourt Brace 1999.
- Dewey J. *Human Nature and Conduct: An Introduction to Social Psychology*. New York: Carlton House 1922.
- Fodor JA: *The Language of Thought*. New York: Crowell 1975.
- Habermas J, Dews P: *Autonomy and Solidarity: Interviews with Jürgen Habermas*. London: Verso 1986.
- Harrington A: *Medicine, Mind, and the Double Brain: A Study in Nineteenth-Century Thought*. Princeton, NJ: Princeton University Press 1987.
- Huber G: *Cerveau et Psychisme Humains: Quelle Ethique? [Human Brain: Which Ethics?]* Paris: Association Decartes and J. Libbey Eurotext 1996.
- Kane R: *The Significance of Free Will*. New York: Oxford University Press 1996.
- Luria AR: *The Man with a Shattered World: The History of a Brain Wound*. New York: Basic Books 1972.
- Minsky M: *The Society of Mind*. New York: Simon and Schuster 1986.
- Nagel T: *Equality and Partiality.* Oxford and New York: Oxford University Press 1991.
- Pylyshyn ZW: *Computation and Cognition: Toward a Foundation for Cognitive Science*. Cambridge, Mass.: MIT Press 1984.
- Rolls ET: *The Brain and Emotion.* Oxford: Oxford University Press 1999.
- Taylor C: *Sources of the Self: The Making of Modern Identity.* Cambridge, Mass.: Harvard University Press 1989.

Since 2002, neuroscientific studies of moral thought, emotions and behaviors have become known as – and a part of – the relatively new discipline of neuroethics [2]. As a field, neuroethics’ focus is not limited to studies of neural bases of morality, but also centers upon those ethical issues that are fostered by neuroscientific research and its various implications and applications in clinical medicine and the public sphere. Thus, as the tools and techniques of neuroscience become more sophisticated and precise, the questions raised by neuroscience and neuroethics may be equally, or even more pressing as those answered [3]. How can –and will–the brain sciences inform concepts of morality, ethics and law? Will understanding the structure and functions of brain networks and processes involved in social interactions, emotions and behaviors alter constructs of “free will,” culpability, and responsibility? Can neuroscientific information provide a basis for guiding how we should behave, either as individuals or as actors-in-community? Will the brain sciences foster a “new ethics” of neuroethics, and if so, how might these new ideas–and perhaps ideals–comport with long held traditions and norms of morality and ethics on an ever more pluralistic world stage?

The late William Safire concluded his introductory remarks to the 2002 Dana Foundation conference “Neuroethics –Mapping the Field” by congratulating the attendees for tackling “…the challenge of carving out a new territory for an old philosophical discipline” [4] by examining the neural mechanisms of morality. The following bibliography reflects this challenging “new territory”, as presented in published works from 2002–2013. These works are experimental, empirical, and/or hypothetical. In some cases the position is inquisitive, in others speculative, and in others a critical perspective is taken (of approaches used to exemplify and study ethical dilemmas, of the prior and current descriptions of psychological processes of human relations, and of concepts of morality and ethics, more generally).

## Methods

Methods for systematically searching relevant literature devoted to neuroethics are identical to those utilized in Part 1 of this bibliography [5]. Search strategies utilizing MeSH (Medical Subject Headings: http://www.ncbi.nlm.nih.gov/mesh/) indexing terms were used for generating bibliographies from PubMed and National Library of Medicine (NLM) Catalog. MeSH includes ethics-related terms developed for BIOETHICSLINE, a specialty database devoted to bioethical issues produced for NLM by the Kennedy Institute of Ethics from 1975–2000. Other databases were searched using descriptors specific to those databases. The searches were limited to work published from 2002 to 2013.

The following databases were searched to produce this bibliography:

1. PubMed (http://pubmed.gov):
  Search Strategy: (morals[majr:noexp] AND (neurosciences/ethics[majr:noexp] OR cognitive science/ethics[majr] OR brain[majr:noexp]))
2. The NLM Catalog (http://www.ncbi.nlm.nih.gov/nlmcatalog):
  Search Strategy: (morals [majr:noexp] AND (neurosciences/ethics[majr:noexp] OR cognitive science/ethics[majr] OR brain[majr:noexp]))
3. Academic Search Premier:
  Search Strategy: TX morality AND SU neurosciences AND SU philosophy
4. Proquest Research Library:
  Search Strategy: su (morality) AND su (neurosciences)
5. JSTOR:
  Search Strategy: ab:(moral) AND ab:(neuroscience)
6. WorldCat (http://www.worldcat.org).:
  Search Strategy: “cognitive neuroscience” and “moral and ethical aspects” (as subject phrases)
7. Philosopher's Index:
  Search Strategy: su(moral) AND su(neuroscience)
8. Embase:
  Search Strategy: neuroscience:de AND morality:de
9. BELIT (http://www.drze.de/belit/).:
  Search Strategy: neurosciences* [subject keywords] and morality*[subject keywords]
10. Web of Knowledge/Web of Science (WoS):
  Search Strategy: [topic] morality neurosciences
11. Digital Public Library of America (DPLA) (http://dp.la/):
  Search Strategy: brain moral
12. Directory of Open Access Journals (DOAJ) (http://www.doaj.org/):
  Search Strategy: [search all] moral neurosciences
13. Hathi Trust Digital Library (http://www.hathitrust.org/):
  [any of these words] morality moral in Subject AND
  [any of these words] neurosciences brain cognitive in Subject
14. European Library (http://www.theeuropeanlibrary.org/tel4/):
  Search Strategy: [subject] moral AND [subject] brain
15. Internet Archive (http://archive.org/):
  Search Strategy: morality AND brain
16. Globethics.net (http://www.globethics.net/):
  Search Strategy: [keywords] moral AND neurosciences
17. Neuroethics-Wikiography (https://teamweb.uni-mainz.de/fb05/Neuroethics):
  Search Strategy: moral

As previously noted [5], open access bioethics’ journals not contained in the Directory of Open Access Journals (DOAJ) were individually accessed and searched; these included:

1. *Journal of Ethics and Social Philosophy* from the University of Southern California http://www.jesp.org/);
2. *Journal of Mental Health Ethics* from McMaster University (http://www.jemh.ca/);
3. *Journal of Practical Ethics* (http://www.jpe.ox.ac.uk/) from the Oxford Uehiro Centre for Practical Ethics at the University of Oxford; and
4. *Philosophers’ Imprint* from the University of Michigan (http://www.philosophersimprint.org/).

As in Part 1 of this bibliography [5], the *RefWorks* citation manager program was utilized to eliminate duplicate reference citations.

## Results

The following reference citations provide a listing of 397 articles, 65 books, and 52 book chapters that afford (1) empirical/experimental studies, overviews, and reviews of neural substrates and mechanisms involved in morality and ethics, and/or (2) reflections upon such studies and their implications.

- Abend G: **Thick concepts and the moral brain.***Euro J Soc* 2011, **52**(1):143–172. doi:10.1017/s0003975611000051.
- Abend G: **What the science of morality doesn’t say about morality.***Philos Soc Sci* 2013, **43**(2):157–200. doi:10.1177/0048393112440597.
- Adolphs R: **The social brain: neural basis of social knowledge.***Annu Rev Psychol* 2009, **60:** 693:716. doi:10.1146/annurev.psych.60.110707.163514.
- Adolphs R: **Cognitive neuroscience of human social behaviour.***Nat Rev Neurosci* 2003, **4**(3): 165–178. doi:10.1038/nrn1056.
- Agar N: **Still afraid of needy post-persons.***J Med Ethics* 2013, **39**(2):81–83. doi:10.1136/medethics-2012-101095.
- Agar N: **Why is it possible to enhance moral status and why doing so is wrong?***J Med Ethics* 2013, **39**(2):67–74. doi:10.1136/medethics-2012-100597.
- Ainslie G: **Précis of breakdown of will.***Behav Brain Sci* 2005, **28**(5):635–673. doi:10.1017/S0140525X05000117.
- Al-Delaimy WK: **Ethical concepts and future challenges of neuroimaging: an Islamic perspective.***Sci Eng Ethics* 2012, **18**(3):509–518. doi:10.1007/s11948-012-9386-3.
- Árnason G: **Neuroscience, free will and moral responsibility.***TRAMES-J Humanit Soc* 2011, **15**(2):147–155. doi:10.3176/tr.2011.2.03.
- Árnason G: **Neuroimaging, uncertainty, and the problem of dispositions.***Camb Q Healthc Ethics* 2010, **19**(2):188–195. doi:10.1017/S0963180109990454.
- Avram M et al.: **Neurofunctional correlates of esthetic and moral judgments.***Neurosci Lett* 2013, **534**:128–132. doi:10.1016/j.neulet.2012.11.053.
- Azzone GF: **The biological foundations of culture and morality.***Rendiconti Lincei* 2008, **19 (2):**189**–**204.doi:10.1007/s12210-008-0011-y.
- Baertschi B: **Neurosciences et neuroéthique: qui se ressemble s’assemble.***Rev Med Suisse* 2005, **1**(34):2225–2229.
- Baertschi B: **Neurosciences et responsabilité morale: un argument en faveur du compatibilisme.***Revue de Theologie et de Philosophie* 2011, **143**(3):257–272.
- Bahnemann M et al.: **Sociotopy in the temporoparietal cortex: common versus distinct processes.***Soc Cogn Affect Neurosci* 2010, **5**(1):48–58. doi:10.1093/scan/nsp045.
- Banja J: **Virtue essentialism, prototypes, and the moral conservative opposition to enhancement technologies: a neuroethical critique.***AJOB Neurosci* 2011, **2**(2):31–38. doi:10.1080/21507740.2011.556918.
- Barandiaran X, Ruiz-Mirazo K: **Modelling autonomy: simulating the essence of life and cognition. Introduction.***Biosystems* 2008, **91**(2):295–304. doi:10.1016/j.biosystems.2007.07.001.
- Barbey AK, Krueger F, Grafman J. **An evolutionarily adaptive neural architecture for social reasoning.***Trends Neurosci* 2009, **32**(12):603–610. doi:10.1016/j.tins.2009.09.001.
- Barraza JA, McCullough ME, Ahmadi S, Zak PJ: **Oxytocin infusion increases charitable donations regardless of monetary resources.***Horm Behav* 2011, **60**(2):148–151. doi:10.1016/j.yhbeh.2011.04.008.
- Bartels DM: **Principled moral sentiment and the flexibility of moral judgment and decision making.***Cognition* 2008, **108**(2):381–417. doi:10.1016/j.cognition.2008.03.001.
- Basile B et al.: **Deontological and altruistic guilt: evidence for distinct neurobiological substrates.***Hum Brain Mapp* 2011, **32**(2):229–239. doi:10.1002/hbm.21009.
- Bellino RM: **Free will in the eye of neuroscientific reductionism: the need for a philosophical foundation of moral neuropsychology.***History & Philosophy of Psychology* 2009, **11**(1):12–16.
- Benedetti F et al.: **Neural and genetic correlates of antidepressant response to sleep deprivation: a functional magnetic resonance imaging study of moral valence decision in bipolar depression.***Arch Gen Psychiatry* 2007, **64**(2):179–187. doi:10.1001/archpsyc.64.2.179.
- Berker S: **The normative insignificance of neuroscience.***Philos Public Aff* 2009, **37**(4):293–329. doi:10.1111/j.1088-4963.2009.01164.x.
- Berns GS, Atran S: **The biology of cultural conflict.***Philos Trans R Soc Lond B Biol Sci* 2012, **367**(1589):633–639. doi:10.1098/rstb.2011.0307.
- Berns GS et al.: **Neurobiological correlates of social conformity and independence during mental rotation.***Biol Psychiatry* 2005, **58**(3):245–253. doi:10.1016/j.biopsych.2005.04.012.
- Berns GS et al.: **The price of your soul: neural evidence for the non-utilitarian representation of sacred values.***Philos Trans R Soc Lond B Biol Sci* 2012, **367**(1589):754–762. doi:10.1098/rstb.2011.0262.
- Berthoz S et al.: **Affective response to one’s own moral violations.***Neuroimage* 2006, **31**(2):945–950. doi:10.1016/j.neuroimage.2005.12.039.
- Berthoz S, Armony JL, Blair RJ, Dolan RJ: **An fMRI study of intentional and unintentional (embarrassing) violations of social norms.***Brain* 2002, **125** (Pt. 8):1696–1708. doi:10.1093/brain/awf190.
- Bertschinger N, Olbrich E, Ay N, Jost J: **Autonomy: an information theoretic perspective.***Biosystems* 2008, **91**(2):331–345.doi:10.1016/j.biosystems.2007.05.018.
- Bird S: **Ethics on the brain.***Science & Spirit* 2006, **17**(4):65–67.
- Blair RJR: **The amygdala and ventromedial prefrontal cortex in morality and psychopathy.***Trends Cogn Sci* 2007, **11**(9):387–392. doi:10.1016/j.tics.2007.07.003.
- Blair J et al.: **Neuro-cognitive systems involved in morality.***Philosophical Explorations: An International Journal for the Philosophy of Mind and Action* 2006, **9**(1):13–27. doi:10.1080/13869790500492359.
- Boden MA: **Autonomy: what is it? Introduction.***Biosystems* 2008, **91**(2):305–308. doi:10.1016/j.biosystems.2007.07.003.
- Bok H: **The implications of advances in neuroscience for freedom of the will.***Neurotherapeutics* 2007, **4**(3):555–559. doi:10.1016/j.nurt.2007.04.001.
- Bonete E: **Neuroethics in Spain: neurological determinism or moral freedom?***Neuroethics* 2013, **6**(1):225–232. doi:10.1007/s12152-012-9151-y.
- Borg JS et al.: **Consequences, action, and intention as factors in moral judgments: an fMRI investigation.***J Cogn Neurosci* 2006, **18**(5):803–817. doi:10.1162/jocn.2006.18.5.803.
- Borg JS, Sinnott-Armstrong W, Calhoun VD, Kiehl KA: **Neural basis of moral verdict and moral deliberation.***Soc Neurosci* 2011, **6**(4):398–413. doi:10.1080/17470919.2011.559363.
- Borg JS, Lieberman D, Kiehl KA: **Infection, incest, and iniquity: investigating the neural correlates of disgust and morality.***J Cogn Neurosci* 2008, **20**(9):1529–1546. doi:10.1162/jocn.2008.20109.
- Boyd GW: **The body, its emotions, the self, and consciousness.***Perspect Biol Med* 2012, **55**(3):362–377. doi:10.1353/pbm.2012.0031.
- Brosch T, Sander D: **Neurocognitive mechanisms underlying value-based decision-making: from core values to economic value.***Front Hum Neurosci* 2013, **7**:398. doi:10.3389/fnhum.2013.00398.
- Brosnan SF: **An evolutionary perspective on morality.***Journal of Economic Behavior & Organization* 2011, **77**(1):23–30. doi:10.1016/j.jebo.2010.04.008.
- Bruni T: **Ventromedial prefrontal cortex lesions and motivational internalism.***AJOB Neurosci* 2012, **3**(3):19–23. doi:10.1080/21507740.2012.694389.
- Bruni T: **Neuroscience and moral reliability.***AJOB Neurosci* 2011, **2**(2):15–17. doi:10.1080/21507740.2011.559913.
- Buford C, Allhoff F: **Neuroscience and metaphysics.***Am J Bioeth* 2005, **5**(2):34–36. doi:10.1080/15265160590960258.
- Buford C, Allhoff F: **Neuroscience and metaphysics (redux**). *Am J Bioeth* 2007, **7**(1):58–60. doi:10.1080/15265160601064272.
- Buller T: **Morality in a blur.***Am J Bioeth* 2008, **8**(5):21–23. doi:10.1080/15265160802180018.
- Buller T: **Rationality, responsibility, and brain function.***Camb Q Healthc Ethics* 2010, **19**(2):196–204. doi:10.1017/S0963180109990466.
- Burgdorf J, Pankseep J: **The neurobiology of positive emotions.***Neurosci Biobehav Rev* 2006, **30**(2):173–187. doi:10.1016/j.neubiorev.2005.06.001.
- Burns K, Bechara A: **Decision making and free will: a neuroscience perspective.***Behav Sci Law* 2007, **25**(2):263–80. doi:10.1002/bsl.751.
- Bzdok D et al.: **Parsing the neural correlates of moral cognition: ALE meta-analysis on morality, theory of mind, and empathy.***Brain Struct Funct* 2012, **217**(4):783–796. doi:10.1007/s00429-012-0380-y.
- Cáceda R et al.: **Mode of effective connectivity within a putative neural network differentiates moral cognitions related to care and justice ethics.***PLoS One* 2011, **6**(2):e14730. doi:10.1371/journal.pone.0014730.
- Cacioppo JT et al. **Just because you're imaging the brain doesn't mean you can stop using your head: a primer and set of first principles.***J Pers Soc Psychol* 2003, **85**(4):650–661. doi:10.1037/0022-3514.85.4.650.
- Camps V: **Neuronas y valores.***Rev Neurol* 2013, **57**(5):230–234.
- Canli T, Amin Z: **Neuroimaging of emotion and personality: scientific evidence and ethical considerations.***Brain Cogn* 2002, **50**(3):414–431.
- Casebeer WD: **Moral cognition and its neural constituents.***Nat Rev Neurosci* 2003, **4**(10):840–846. doi:10.1038/nrn1223.
- Casebeer WD, Churchland PS: **The neural mechanisms of moral cognition: a multiple-aspect approach to moral judgment and decision-making.***Biol Philos* 2003, **18**(1):169–194. doi:10.1023/A:1023380907603.
- Caspers S et al.: **Moral concepts set decision strategies to abstract values.***PLoS One* 2011, **6**(4):e18451. doi:10.1371/journal.pone.0018451.
- Chambon V et al.: **An online neural substrate for a sense of agency.***Cereb Cortex* 2013, **23**(5):1031–1037. doi:10.1093/cercor/bhs059.
- Chaminade T, Decety J: **Leader or follower? Involvement of the inferior parietal lobule in agency.***Neuroreport* 2002, **13**(15):1975–1978. doi:10.1097/00001756-200210280-00029.
- Chang LJ, Smith A, Dufwenberg M, Sanfey AG: **Triangulating the neural, psychological, and economic bases of guilt aversion.***Neuron* 2011, **70**(3):560–572. doi:10.1016/j.neuron.2011.02.056.
- Charmetant E: **Contemporary naturalism and human ontology: towards a different essentialism.***Forum Philosophicum: International Journal for Philosophy* 2011, **16**(1):59–72.
- Check E: **Ethicists urge caution over emotive power of brain scans.***Nature* 2005, **435**(7040):254–255. doi:10.1038/435254a.
- Cheshire WP: **The origami brain: from neural folds to neuroethics.***Ethics Med* 2011, **27**(2):79–83.
- Cheshire WP: **Can grey voxels resolve neuroethical dilemmas?***Ethics Med* 2007, **23**(3):135–140.
- Chiong W: **The self: from philosophy to cognitive neuroscience.***Neurocase* 2011, **17**(3):190–200. doi:10.1080/13554794.2010.532808.
- Christensen JF, Gomila A: **Moral dilemmas in cognitive neuroscience of moral decision-making: a principled review.***Neurosci Biobehav Rev* 2012, **36**(4):1249–1264. doi:10.1016/j.neubiorev.2012.02.008.
- Churchland PM: **Into the brain: where philosophy should go from here.***Topoi-Int Rev Philos* 2006, **25**(1–2):29–32. doi:10.1007/s11245-006-0024-z.
- Churchland PS: **The impact of neuroscience on philosophy.***Neuron* 2008, **60**(3):409–411. doi:10.1016/j.neuron.2008.10.023.
- Churchland PS: **Neurophilosophy: the early years and new directions.***Funct Neurol* 2007, **22**(4):185–195.
- Churchland PS: **Free will matters.***AJOB Neurosci* 2011, **2**(3):1–2. doi:10.1080/21507740.2011.588905.
- Ciaramelli E, di Pellegrino G: **Ventromedial prefrontal cortex and the future of morality.***Emotion Review* 2011, **3**(3):308–309. doi:10.1177/1754073911402381.
- Ciaramelli E, Sperotto RG, Mattioli F, di Pellegrino G: **Damage to the ventromedial prefrontal cortex reduces interpersonal disgust.*** Soc Cogn Affect Neurosci* 2013, **8**(2):171–180. doi:10.1093/scan/nss087.
- Ciaramidaro A, et al.: **The intentional network: how the brain reads varieties of intentions.***Neuropsychologia* 2007, **45**(13):3105–3113. doi:10.1016/j. neuropsychologia.2007.05.011.
- Cikara M, Farnsworth RA, Harris LT, Fiske ST: **On the wrong side of the trolley track: neural correlates of relative social valuation.***Soc Cogn Affect Neurosci* 2010, **5**(4):404–413. doi:10.1093/scan/nsq011.
- Claes SJ: **The free brain?***Tijdschr Psychiatr* 2011, **53**(7):389–391.
- Clark TW: **Holding mechanisms responsible.***Med Ethics (Burlingt Mass)* 2006, **13**(3):10–11.
- Cole Wright J, Cullum J, Schwab N: **The cognitive and affective dimensions of moral conviction: implications for attitudinal and behavioral measures of interpersonal tolerance.***Pers Soc Psychol Bull* 2008, **34**(11):1461–1476. doi:10.1177/0146167208322557.
- Conway P, Gawronski B: **Deontological and utilitarian inclinations in moral decision making: a process dissociation approach.***J Pers Soc Psychol* 2013, **104**(2):216–235. doi:10.1037/a0031021.
- Cosmides L, Tooby J, Fiddick L, Bryant GA: **Detecting cheaters.***Trends Cogn Sci* 2005, **9**(11):505–506. doi:10.1109/msp.2011.28.
- Costa AN: **Images of difficulty.***IIOAB Journal* 2013, **4**(3):3–8.
- Crockett MJ, Clark L, Hauser MD et al.: **Serotonin selectively influences moral judgment and behavior through effects on harm aversion.***Proc Natl Acad Sci* USA 2010, **107**(40):17433–17438. doi:10.1073/pnas.1009396107.
- Cromby J, Newton T, Williams SJ: **Neuroscience and subjectivity.***Subjectivity* 2011, **4**:215–226. doi:10.1057/sub.2011.13.
- Culotta E: **Neuroscience. Brain stimulation sparks ‘Machiavellian’ choices.***Science* 2013, **342**(6154):25. doi:10.1126/science.342.6154.25.
- Cushman F, Knobe J, Sinnott-Armstrong W: **Moral appraisals affect doing/allowing judgments.***Cognition* 2008, **108**(1):281–289. doi:10.1016/j.cognition.2008.02.005.
- Cushman F: **Action, outcome, and value: a dual-system for morality.***Pers Soc Psychol Rev* 2013, **17**(3):273–292. doi:10.1177/1088868313495594.
- Cushman F, Young L, Hauser M: **The role of conscious reasoning and intuition in moral judgment: testing three principles of harm.***Psychol Sci* 2006, **17**(12):1082–1089. doi:10.1111/j.1467-9280.2006.01834.x.
- Cushman F, Greene JD: **Finding faults: how moral dilemmas illuminate cognitive structure.***Soc Neurosci* 2012, **7**(3):269–279. doi:10.1080/17470919.2011.614000.
- Damasio A: **Neuroscience and ethics: intersections.***Am J Bioeth* 2007, **7**(1):3–7. doi:10.1080/1526516060103910.
- da Rocha AF, Rocha FT, Massad E: **Moral dilemma judgment revisited: a Loreta analysis.***J Behav Brain Sci* 2013, **3**(8):624–640. doi:10.4236/jbbs.2013.38066.
- da Rocha AC, Bergareche AM: **Wired for autonomy.***Am J Bioeth* 2008, **8**(5):23–25. doi:10.1080/15265160802180042.
- Dawes CT et al.: **Neural basis of egalitarian behavior.***Proc Natl Acad Sci* 2012, **109**(17):6479–6483. doi:10.1073/pnas.1118653109.
- de Achával D et al.: **Activation of brain areas concerned with social cognition during moral decisions is abnormal in schizophrenia patients and unaffected siblings.***J Psychiatr Res* 2013, **47**(6):774–782. doi:10.1016/j.jpsychires.2012.12.018.
- Dean R: **Does neuroscience undermine deontological theory?***Neuroethics* 2010, **3**(1):43–60. doi:10.1007/s12152-009-9052-x.
- Decety J, Michalska KJ, Kinzler KD: **The developmental neuroscience of moral sensitivity.***Emot Rev* 2011, **3**(3):305–307. doi:10.1177/1754073911402373.
- Decety J, Howard LH: **The role of affect in the neurodevelopment of morality.***Child Dev Perspect* 2013, **7**(1):49–54. doi:10.1111/cdep.12020.
- Decety J, Cacioppo S: **The speed of morality: a high-density electrical neuroimaging study.***J Neurophysiol* 2012, **108**(11):3068–3072. doi:10.1152/jn.00473.2012.
- Decety J, Michalska, KJ: **Neurodevelopmental changes in the circuits underlying empathy and sympathy from childhood to adulthood.***Dev Sci* 2010, **13**(6):886–899. doi:10.1111/j.1467-7687.2009.00940.x.
- Decety J, Porges EC: **Imagining being the agent of actions that carry different moral consequences: an fMRI study.***Neuropsychologia* 2011, **49**(11):2994–3001. doi:10.1016/j.neuropsychologia.2011.06.024.
- Decety J: **The neurodevelopment of empathy in humans.***Dev Neurosci* 2010, **32**(4):257–267. doi:10.1159/000317771.
- Decety J, Michalska KJ, Kinzler KD:** The contribution of emotion and cognition to moral sensitivity: a neurodevelopmental study.***Cereb Cortex* 2012, **22**(1):209–220. doi:10.1093/cercor/bhr111.
- Dedeke A: **A cognitive-intuitionist model of moral judgment***. J Bus Ethics* 2013 (online). doi:10.1007/s10551-013-1965-y.
- de Jong BM: **Neurology of widely embedded free will.***Cortex* 2011, **47**(10):1160–1065. doi:10.1016/j.cortex.2011.06.011.
- Delgado MR, Frank RH, Phelps EA: **Perceptions of moral character modulate the neural systems of reward during the trust game.***Nat Neurosci* 2005, **8**(11):1611–1618. doi:10.1038/nn1575.
- Demirtas-Tatlidede A, Schmahmann JD: **Morality: incomplete without the cerebellum**? *Brain* 2013, **136**(8):e244. doi:10.1093/brain/awt070.
- de Quervain DJ et al.: **The neural basis of altruistic punishment.***Science* 2004, **305**(5688):1254–1258. doi:10.1126/science.1100735.
- de Waal F: **The animal roots of human morality.***New Sci* 2006, **192**(2573):60–61. doi: 10.1016/S0262-4079(06)60737-9.
- Díaz JL: **The psychobiology of aggression and violence: bioethical implications.***Int Soc Sci J* 2010, **61**(200–201):233–245. doi:10.1111/j.1468-2451.2011.01760.x.
- Di Francesco M, Motterlini M, Colombo M: **In search of the neurobiological basis of decision making: explanation, reduction and emergence.***Funct Neurol* 2007, **22**(4):197–204.
- Di Francesco M: **Neurofilosofia, naturalismo e statuto dei giudizi morali.***Etica e Politica* 2007, **9**(2):126–143.
- Dinh JE, Lord RG: **Current trends in moral research: what we know and where to go from here.***Curr Dir Psychol Sci* 2013, **22**(5):380–385. doi:10.1177/0963721413486147.
- Di Paolo EA, Iizuka H: **How (not) to model autonomous behavior.***Biosystems* 2008, **91**(2):409–423. doi:10.1016/j.biosystems.2007.05.016.
- Drabant EM et al.: **Experiential, autonomic, and neural responses during threat anticipation vary as a function of threat intensity and neuroticism.***Neuroimage* 2011, **55**(1):401–410. doi:10.1016/j.neuroimage.2010.11.040.
- Dulac C, Rizzolatti G: **Neurobiology of behavior.***Curr Opin Neurobiol* 2009, **19**(6):634–636. doi:10.1016/j.conb.2009.10.009.
- Dupoux E, Jacob P: **Universal moral grammar: a critical appraisal.***Trends Cogn Sci* 2007, **11**(9):373–378. doi:10.1016/j.tics.2007.07.001.
- Dwyer S, Hauser MD: **Dupoux and Jacob’s moral instincts: throwing out the baby, the bathwater and the bathtub.***Trends Cogn Sci* 2008, **12**(1):1–2. doi:10.1016/j.tics.2007.10.006.
- Ellemers N: **Is it better to be moral than smart? The effects of morality and competence norms on the decision to work at a group status improvement.***J Pers Soc Psychol* 2008, **95**(6):1397–1410. doi:10.1037/a0012628.
- Epstein M: **Critique of moral judgment: sociology versus neuroscience.***AJOB Neurosci* 2011, **2**(2):26–28. doi:10.1080/21507740.2011.559916.
- Erickson SK, Felthous AR: **Introduction to this issue: the neuroscience and psychology of moral decision making and the law.***Behav Sci Law* 2009, **27**(2):119–121. doi:10.1002/bsl.858.
- Evers K: **Towards a philosophy for neuroethics: an informed materialist view of the brain might help to develop theoretical frameworks for applied neuroethics.***EMBO Rep* 2007, **8**:S48-S51. doi:10.1038/sj.embor.7401014.
- Farah MJ, Murphy N: **Neuroscience and the soul.***Science* 2009, **323**(5918):1168. doi:10.1126/science.323.5918.1168a.
- Farah MJ, Heberlein AS: **Personhood and neuroscience: naturalizing or nihilating?***Am J Bioeth* 2007, **7**(1):37–48. doi:10.1080/15265160601064199.
- Farrer C, Frith CD: **Experiencing oneself vs. another person as being the cause of an action: the neural correlates of the experience of agency.***Neuroimage* 2002, **15**(3):596–603. doi:10.1006/nimg.2001.1009.
- Fiddick L: **Domains of deontic reasoning: resolving the discrepancy between the cognitive and moral reasoning literatures.***QJ Exp Psychol A* 2004, **57**(3):447–474. doi:10.1080/02724980343000332.
- Figdor C: **What is the “cognitive” in cognitive neuroscience?***Neuroethics* 2013, **6**(1):105–114. doi:10.1007/s12152-012-9157-5.
- Figueroa G: **Neuroethics: reflections on the latent principles of morals in medicine.***Rev Med Chil* 2012, **140**(8):1078–1084. doi:10.4067/S0034-98872012000800018.
- Finger EC et al.: **Caught in the act: the impact of audience on the neural response to morally and socially inappropriate behavior.***NeuroImage* 2006, **33**(1):414–421. doi:10.1016/j.neuroimage.2006.06.011.
- Fins JJ: **Lessons from the injured brain: a bioethicist in the vineyards of neuroscience.***Camb Q Healthc Ethics* 2009, **18**(1):7–13. doi:10.1017/S0963180108090038.
- Fiske ST, Cuddy AJ, Glick P: **Universal dimensions of social cognition: warmth and competence.***Trends Cogn Sci* 2007, **11**(2):77–83. doi:10.1016/j.tics.2006.11.005.
- Fitzpatrick M: **Can morality ever become a science?***Dialogue-J Phi Sigma***54**(2–3):138–154.
- Flanagan O: **One enchanted being: neuroexistentialism and meaning.***Zygon* 2009, **44**(1):41–49. doi:10.1111/j.1467-9744.2009.00984.x.
- Forbes CE, Grafman J: **The role of the human prefrontal cortex in social cognition and moral judgment.***Annu Rev Neurosci* 2010, **33**:299–324. doi:10.1146/annurev-neuro-060909-153230.
- Friedman JH: **Chemical imbalance or moral weakness? Personal responsibility in a time of brain science.***Med Health R I* 2006, **89**(3):88.
- Frost CJ, Lumia AR: **The ethics of neuroscience and the neuroscience of ethics: A phenomenological–existential approach.***Sci Eng Ethics* 2012, **18**(3):457–474. doi:10.1007/s11948-012-9388-1.
- Fumagalli M, Priori A: **Functional and clinical neuroanatomy of morality.***Brain* 2012, **135**(7):2006–2021. doi:10.1093/brain/awr334.
- Fumagalli M et al.: **Conflict-dependent dynamic of subthalamic nucleus oscillations during moral decisions.***Soc Neurosci* 2011, **6**(3):243–256. doi:10.1080/17470919.2010.515148.
- Fumagalli M et al.: **Gender-related differences in moral judgments.***Cogn Process* 2010, **11**(3):219–226. doi:10.1007/s10339-009-0335-2.
- Fumagalli M et al.: **Brain switches utilitarian behavior: does gender make a difference?***PloS One* 2010, **5**(1):e8865. doi:10.1371/journal.pone.0008865.
- Funayama M, Mimura M: **Orbitofrontal cortex and morality.***Brain and Nerve/Shinkei Kenkyu no Shinpo* 2012, **64**(10):1121–1129.
- Funk CM, Gazzaniga MS: **The functional brain architecture of human morality.***Curr Opin Neurobiol* 2009, **19**(6):678–681. doi:10.1016/j.conb.2009.09.011.
- Gerber B: **Is there a neurobiology of the free will?***EMBO Rep* 2011, **13**(1):17–19. doi:10.1038/embor.2011.229.
- Gerrans P, Kennett J: **Neurosentimentalism and moral agency.***Mind* 2010, **119**(475):585–614. doi:10.1093/mind/fzq037.
- Gert B: **Neuroscience and morality.***Hastings Cent Rep* 2012, **42**(3):22–28. doi:10.1002/hast.36.
- Giligorov N: **Determinism and advances in neuroscience.***Virtual Mentor* 2012, **14**(6):489–493. doi:10.1001/virtualmentor.2012.14.6.msoc1-1206.
- Gillett GR: **The subjective brain, identity, and neuroethics.***Am J Bioeth* 2009 **9**(9):5–13. doi:10.1080/15265160903090058.
- Glannon W: **Free will and moral responsibility in the age of neuroscience.***Med Ethics (Burlingt Mass)* 2006, **13**(2):1–2.
- Gleichgerrcht E et al.: **The role of social cognition in moral judgment in frontotemporal dementia.***Soc Neurosci* 2011, **6**(2):113–122. doi:10.1080/17470919.2010.506751.
- Glenn AL, Raine A, Schug RA: **The neural correlates of moral decision-making is psychopathy.***Mol Psychiatry* 2009, **14**(1):5–6. doi:10.1038/mp.2008.104.
- Gligorov N: **Determinism and advances in neuroscience.***Virtual Mentor* 2012, **14**(6):489–493. doi:10.1001/virtualmentor.2012.14.6.msoc1-1206.
- Glimcher PW, Rustichini A: **Neuroeconomics: the consilience of brain and decision.***Science* 2004, **306**(5695):447–452. doi:10.1126/science.1102566.
- Gold I, Kirmayer LJ: **Neuroscience as cultural intervention: reconfiguring the self as moral agent.***AJOB Neurosci* 2010, **1**(4):53–55. doi:10.1080/21507740.2010.514884.
- Gomila A, Calvo P: **Understanding brain circuits and their dynamics.***Behav Brain Sci* 2010, **33**(4):274–275. doi:10.1017/S0140525X10001238.
- Goodenough U, Deacon TW: **From biology to consciousness to morality.***Zygon* 2003, **38**(4):801–819. doi:10.1111/j.1467-9744.2003.00540.x.
- Grahm J, Nosek BA, Haidt J, IyerR et al.: **Mapping the moral domain.***J Pers Soc Psychol* 2011, **101**(2):366–385. doi:10.1037/a0021847.
- Green S et al.: **Selective functional integration between anterior temporal and distinct fronto-mesolimbic regions during guilt and indignation.***Neuroimage* 2010, **52**(4):1720–1726. doi:10.1016/j.neuroimage.2010.05.038.
- Greene JD et al.: **The neural bases of cognitive conflict and control in moral judgment.***Neuron* 2004, **44**(2):389–400. doi:10.1016/j.neuron.2004.09.027.
- Greene JD et al.: **Cognitive load selectively interferes with utilitarian moral judgment.***Cognition* 2008, **107**(3):1144–1154. doi:10.1016/j.cognition.2007.11.00.4.
- Greene J: **Opinion: from neural ‘is’ to moral ‘ought’: what are the moral implications of neuroscientific moral psychology?***Nat Rev Neurosci* 2003, **4**:846–850. doi:10.1038/nrn1224.
- Greene J, Haidt J: **How (and where) does moral judgment work**? *Trends Cog Sci* 2002, **6**(12):517–523. doi:10.1016/s1364-6613(02)02011-9.
- Greene JD**: Emotion and morality: A tasting menu.***Emot Rev* 2011, **3**(3):1–3. doi:10.1177/1754073911409629.
- Greene JD: **Why are VMPFC patients more utilitarian**? **A dual-process theory of moral judgment explains.***Trends Cogn Sci* 2007, **11**(8):322–323. doi:10.1016/j.tics.2007.06.004.
- Greene JD: **Dual-process morality and the personal/impersonal distinction: a reply to McGuire, Langdon, Coltheart, and Mackenzie.***J Exp Soc Psychol* 2009, **45**(3):581–584. doi:10.1016/j.jesp.2009.01.003.
- Greene JD et al.: **Pushing moral buttons: the interaction between personal force and intention in moral judgment.***Cognition* 2009, **111**(3):364–371. doi:10.1016/j.cognition.2009.02.001.
- Greenspan RJ: **Biological indeterminacy.***Sci Eng Ethics* 2012, **18**(3):447–452. doi:10.1007/s11948-012-9379-2.
- Güroğlu B, van den Bos W, Rombouts SA, Crone EA: **Unfair? It depends: a neural correlates of fairness in social context.***Soc Cogn Affect Neurosci* 2010, **5**(4):414–423. doi:10.1093/scan/nsq013.
- Haidt J, Morris JP: **Finding the self in self-transcendent emotions.***Proc Natl Acad Sci USA* 2009, **106**(19):7687–7688. doi:10.1073/pnas.0903076106.
- Haidt J: **The new synthesis in moral psychology.***Science* 2007, **316**(5827):998–1002. doi:10.1126/science.1137651.
- Haidt J, Joseph C: **Intuitive ethics: how innately prepared intuitions generate culturally variable virtues.***Daedalus* 2004, **133**(4):55–66. doi:10.1162/0011526042365555.
- Haidt J: **Morality.***Perspect Psychol Sci* 2008, **3**(1):65–72. doi:10.1111/j.1745-6916.2008.00063.x.
- Harenski CL, Antonenko O, Shane MS, Kiehl KA: **Gender differences in neural mechanisms underlying moral sensitivity.***Soc Cogn Affect Neurosci* 2008, **3**(4):313–321. doi:10.1093/scan/nsn026.
- Harenski CL, Hamann S: **Neural correlates of regulating negative emotions related to moral violations.***Neuroimage* 2006, **30**(1):313–324. doi:10.1016/j.neuroimage.2005.09.034.
- Harenski CL, Antonenko O, Shane MS, Kiehl KA: **A functional imaging investigation of moral deliberation and moral intuition.***Neuroimage* 2010, **49**(3):2707–2716. doi:10.1016/j.neuroimage.2009.10.062.
- Hayashi A et al.: **Neural correlates of forgiveness for moral transgressions involving deception.***Brain Res* 2010, **1332**:90–99. doi:10.1016/j.brainres.2010.03.045.
- Häyry M: **Neuroethical theories.***Camb Q Healthc Ethics* 2010, **19**(2):165–178. doi:10.1017/S0963180109990430.
- Heekeren HR et al.: **Influence of bodily harm on neural correlates of semantic and moral decision-making.***Neuroimage* 2005, **24**(3):887–897. doi:10.1016/j.neuroimage.2004.09.026.
- Heekeren HR et al.: **An fMRI study of simple ethical decision-making.***Neuroreport* 2003, **14**(9):1215–1219. doi:10.1097/00001756-200307010-00005.
- Heinzelmann N, Ugazio G, Tobler PN: **Pragmatic implications of empirically studying moral decision-making.***Front Neurosci* 2012, **6**:94. doi:10.3389/fnins.2012.00094.
- Hsu M, Anen C, Quartz SR: **The right and the good: distributive justice and neural encoding of equity and efficiency.***Science* 2008, **320**(5879):1092–1095. doi:10.1126/science.1153651.
- Ikegami T, Suzuki K: **From a homeostatic to homeodynamic self.***Biosystems* 2008, **91**(2):388–400. doi:10.1016/j.biosystems.2007.05.014.
- Illes J**: Empirical neuroethics: can brain imaging visualize human thought? Why is neuroethics interested in such a possibility?***EMBO Rep* 2007, **8**(Suppl 1):S48-S51. doi:10.1038/sj.embor.7401007.
- Jack AI, Dawson AJ, Norr ME: **Seeing human: distinct and overlapping neural signatures associated with two forms of dehumanization.***Neuroimage* 2013, **79**:313–328. doi:10.1016/j.neuroimage.2013.04.109.
- Jedlicka P: **Neuroethics, reductionism, and dualism.***Trends Cogn Sci* 2005, **9**(4):172. doi:10.1016/j.tics.2005.02.010.
- Jirsa J: **Morality as a science?***Filos Cas* 2013, **61**(4):581–590.
- Júnior RM: **Neuroética: o cérebro como órgão da ética e da moral.***Revista Bioética* 2010, **18**(1):109–120.
- Kahane G et al.: **The neural basis of intuitive and counterintuitive moral judgment.** Soc Cogn Affect Neurosci 2012, **7**(4):393–402. doi:10.1093/scan/nsr005.
- Kahane G, Shackel N: **Methodological issues in the neuroscience of moral judgment.***Mind Lang* 2010, **25**(5):561–582. doi:10.1111/j.1468-0017.2010.01401.x.
- Kahn PH Jr: **Mind and morality.***New Dir Child Adolesc Dev* 2004, **103**:73–83. doi:10.1002/cd.99.
- Kamm FM: **Neuroscience and moral reasoning: a note on recent research.***Philos Public Aff* 2009, **37**(4):330–345. doi:10.1111/j.1088-4963.2009.01165.x.
- Kaposy C: **Will neuroscientific discoveries about free will and selfhood change our ethical practices?***Neuroethics* 2009, **2**(1):51–59. doi:10.1007/s12152-008-9020-x.
- Karlsson H: **Psychiatry, neuroscience and free will.***Nord J Psychiatry* 2005, **59**(1):5. doi:10.1080/08039480510018869.
- Kaufman WRP**: Can science determine moral values? a reply to Sam Harris.***Neuroethics* 2012, **5**(1):55–65. doi:10.1007/s12152-010-9096-y.
- Kauppinen A: **Moral internalism and the brain.***Soc Theory Pract* 2008, **34**(1):1–24. doi:10.5840/soctheorpract20083411.
- Kawohl W, Habermeyer E: **Free will: reconciling German civil law with Libet’s neuropsychological studies on the readiness potential.***Behav Sci Law* 2007, **25**(2):309–320. doi:10.1002/bsl.752.
- Keller M, Gummerum M, Wang XT, Lindsey S: **Understanding perspectives and emotions in contract violation: development of deontic and moral reasoning.***Child Dev* 2004, **75**(2):614–635. doi:10.1111/j.1467-8624.2004.00696.x.
- Killen M, Smetana J: **Moral judgment and moral neuroscience: intersections, definitions, and issues.***Child Dev Perspect* 2008, **2**(1):1–6. doi:10.1111/j.1750-8606.2008.00033.x.
- Killen M et al.: **The accidental transgressor: morally-relevant theory of mind.***Cognition* 2011, **119**(2):197–215. doi:10.1016/j.cognition.2011.01.006.
- Killen M, Smetana J: **The biology of morality: human development and moral neuroscience.***Human Development* 2007, **50**(5):241–243. doi:10.1159/000106413.
- Kittay EF: **At the margins of moral personhood.***Ethics* 2005, **116**(1):100–131. doi:10.1086/454366.
- Klosterkötter J: **Subjective experience and neuroscience.***Fortschr Neurol Psychiatr* 2006, **74**(11):619–620. doi:10.1055/s-2006-944286.
- Knabb JJ, Welsh RK, Ziebell JG, Reimer KS: **Neuroscience, moral reasoning, and the law.***Behav Sci Law* 2009, **27**(2):219–236. doi:10.1002/bsl.854.
- Knobe J: **Theory of mind and moral cognition: exploring the connections.***Trends Cogn Sci* 2005, **9**(8):357–359. doi:10.1016/j.tics.2005.06.011.
- Koenigs M et al.: **Damage to the prefrontal cortex increases utilitarian moral judgments.***Nature* 2007, **446**(7138):908–911. doi:10.1038/nature05631.
- Koenigs M: **The neuropsychology of disgust.***Soc Cogn Affect Neurosci* 2013, **8**(2):121–122. doi:10.1093/scan/nss134.
- Koscik TR, Tranel D: **The human amygdala is necessary for developing and expressing normal interpersonal trust.***Neuropsychologia* 2011, **49**(4):602–611. doi:10.1016/j.neuropsychologia.2010.09.023.
- Kosfeld M et al.: **Oxytocin increases trust in human.***Nature* 2005, **435**(7042):673–676. doi:10.1038/nature03701.
- Koster-Hale J, Saxe R, Dungan J, Young LL: **Decoding moral judgments from neural represents of intentions.** Proc Natl Acad Sci USA 2013, **110**(14):5648–5653. doi:10.1073/pnas.1207992110.
- Krawczyk DC: **Contributions of the prefrontal cortex to the neural basis of human decision making.***Neurosci Biobehav Rev* 2002, **26**(6):631–664. doi:10.1016/S0149-7634(02)00021-0.
- Krebs DL, Denton K:**Explanatory limitations of cognitive-developmental approaches to morality.***Psychol Rev* 2006, **113**(3):672–675. doi:10.1037/0033-295x.113.3.672.
- Kvaran T, Nichols S, Sanfey A: **The effect of analytic and experiential modes of thought on moral judgment.***Prog Brain Res* 2013, **202**:187–196. doi:10.1016/B978-0-444-62604-2.00011-3.
- Kvaran T, Sanfey AG: **Toward an integrated neuroscience of morality: the contribution of neuroeconomics to moral cognition.***Top Cog Sci* 2010, **2**(3):579–595.doi:10.1111/j.1756-8765.2010.01086.x.
- Lahat A, Helwig CC, Zelazo PD: **An event-related potential study of adolescents’ and young adults’ judgments of moral and social conventional violations.***Child Dev* 2013, **84**(3):955–969. doi:10.1111/cdev.12001.
- Lampe M: **Science, human nature, and a new paradigm for ethics education.***Sci Eng Ethics* 2012, **18**(3):543–549. doi:10.1007/s11948-012-9373-8.
- Lane JD, Wellman HM, Olson SL, LaBounty J, Kerr DC: **Theory of mind and emotion understanding predict moral development in early childhood.***Br J Dev Psychol* 2010, **28**(4):871–889. doi:10.1348/026151009x483056.
- Leben D: **Cognitive neuroscience and moral decision-making: guide or set aside**? *Neuroethics* 2011, **4**(2):163–174. doi:10.1007/s12152-010-9087-z.
- Lee W, Reeve J: **Self-determined, but not non-self-determined, motivation predicts activations in the anterior insular cortex: an fMRI study of personal agency.***Soc Cogn Affect Neurosci* 2013, **8**(5):538–545. doi:10.1093/scan/nss029.
- Legault L, Inzlicht M: **Self-determination, self-regulation, and the brain: autonomy improves performance by enhancing neuroaffective responsiveness to self-regulation failure.***J Pers Soc Psychol* 2013, **105**(1):123–138. doi:10.1037/a0030426.
- Lev O: **Enhancing the capacity for moral agency.***AJOB Neurosci* 2012, **3**(4):20–22. doi:10.1080/21507740.2012.721462.
- Levy DA: **Neural holism and free will.***Philos Psychol* 2003, **16**(2):205–228. doi:10.1080/09515080307765.
- Levy N: **Cognitive scientific challenges to morality.***Philos Psychol* 2006, **19**(5):567–587. doi:10.1080/09515080600901863.
- Levy N: **Empirically informed moral theory: a sketch of the landscape.***Ethical Theory Moral Pract* 2009, **12**(1):3–8. doi:10.1007/s10677-008-9146-2.
- Levy N: **Rethinking neuroethics in the light of the extended mind thesis.***Am J Bioeth* 2007, **7**(9):3–11. doi:10.1080/15265160701518466.
- Lewis GJ, Kanai R, Bates TC, Rees G: **Moral values are associated with individual differences in regional brain volume.***J Cogn Neurosci* 2012, **24**(8):1657–1663. doi:10.1162/jocn_a_00239.
- Li P, Jia S, Feng T, Liu Q, Suo T, Li H: **The influence of the diffusion of responsibility effect on outcome evaluations: electrophysiological evidence from an ERP study.***Neuroimage* 2010, **52**(4):1727–1733. doi:10.1016/j.neuroimage.2010.04.275.
- Longuenesse B: **“I” and the brain.***Psychol Res* 2012, **76**(2):220–228. doi:10.1007/s00426-011-0382-z.
- Loye D: **The moral brain.***Brain and Mind* 2002, **3**(1):133–150. doi:10.1023/A:1016561925565.
- Lunstroth J, Goldman J: **Ethical intelligence from neuroscience: is it possible**? *AJOB* 2007, **7**(5):18–20. doi:10.1080/15265160701290280.
- Lunstroth J: **No strangers: medicine, neuroscience, and philosophy.***Am J Bioeth* 2008, **8**(1):59–61. doi:10.1080/15265160701839680.
- Luo Q et al.: **The neural basis of implicit moral attitude—an IAT study using event-related MR**I. *Neuroimage* 2006, **30**(4):1449–1457. doi:10.1016/j.neuroimage.2005.11.005.
- Majdandžić J et al.: **The human factor: behavioral and neural correlates of humanized perception in moral decision making.***PLoS One* 2012, **7**(10):e47698. doi:10.1371/journal.pone.0047698.
- Malti T, Gummerum M, Keller M: **Moral emotions and moral cognitions.***Int J Dev Sci* 2008, **2**(3):203–205. doi:10.3233/DEV-2008-2301.
- Margoni F: **Dilemmi e scelte morali, oltre Greene?***BrainFactor* 2013, **5**(3):005.
- Margoni F: **Emozioni diverse per giudizi morali diversi?***BrainFacto*r 2013, **5**(4):010.
- Margoni F: **Fattori irrilevanti e giudizio morale.***BrainFactor* 2013, 5(2):006.
- Markic O: **Nevroetika: vprasanje moralne odgovornosti.***Casopis za Kritiko Znanosti* 2011, **39**(246):15–25, 223, 229.
- McClelland RT: **The pleasures of revenge.***Journal of Mind and Behavior* 2010, **31**(3–4):195–235.
- McGoldrick TA: **The spirituality of human consciousness: a Catholic evaluation of some current neuro-scientific interpretations.***Sci Eng Ethics* 2012, **18**(3):483–501. doi:10.1007/s11948-012-9387-2.
- McGuire J, Langdon R, Coltheart M, Mackenzie C: **A reanalysis of the personal/impersonal distinction in moral psychology research.***J Exp Soc Psychol* 2009, **45**(3):581–584.
- McKaughan DJ: **Voles, vasopressin, and infidelity: a molecular basis for monogamy, a platform for ethics, and more?***Biol Philos* 2012, **27**(4):521–543. doi:10.1007/s10539-011-9303-1.
- Meeks TW, Jeste DV: **Neurobiology of wisdom: a literature overview.***Arch Gen Psychiatry* 2009, **66**(4):355–365. doi:10.1001/archgenpsychiatry.2009.8.
- Mele A: **Free will and neuroscience.***Philos Exch* 2013, **43**(1):3. http://digitalcommons.brockport.edu/phil_ex/vol43/iss1/3.
- Mele A: **Unconscious decisions and free will.***Philos Psychol* 2013, **26**(6):777–789. doi:10.1080/09515089.2012.724395.
- Meloni M: **Moralizing biology: the appeal and limits of the new compassionate view of nature.***Hist Human Sci* 2013, **26**(3):82–106. doi:10.1177/0952695113492163.
- Mende-Siedlecki P, Baron SG, Todorov A: **Diagnostic value underlies asymmetric updating impressions in the morality and ability domains.***J Neurosci* 2013, **33**(50):19406–19415. doi:10.1523/jneurosci.2334-13.2013.
- Mendez MF: **The neurobiology of moral behavior: review and neuropsychiatric implications.***CNS Spectr* 2009, **14**(11):608–620.
- Mercadillo RE, Arias NA: **Violence and compassion: a bioethical insight into their cognitive bases and social manifestations.***Int Soc Sci J* 2010, **61**(200–201):221–232. doi:10.1111/j.1468-2451.2011.01759.x.
- Mercadillo RE, Diaz JL, Barrios FA: **Neurobiología de las emociones morales.***Salud Ment (Mex)* 2007, **30**(3):1–11.
- Mikhail J: **Universal moral grammar: theory, evidence, and the future.***Trends Cogn Sci* 2007, **11**(4):143–152. doi:10.1016/j.tics.2006.12.007.
- Mikhail J: **Emotion, neuroscience, and law: A comment on Darwin and Greene.***Emot Rev* 2011, **3**(3):293–295.doi:10.1177/1754073911406150.
- Miller G: **The roots of morality.***Science* 2008, **320**(5877):734–737. doi:10.1126/science.320.5877.734.
- Mimura M: **Role of orbitofrontal cortex in moral judgment.***Rinsho Shinkeigaku* 2010, **50**(11):1007–1009. doi:10.5692/clinicalneurol.50.1007.
- Mirabella G: **Endogenous inhibition and the neural basis of “free won’t**.” *J Neurosci* 2007, **27**(51):13919–13920. doi:10.1523/jneurosci.4943-07.2007.
- Mograbi GJC: **Neurophilosophical considerations on decision making: pushing-up the frontiers without disregarding their foundations***. Front Neurosci* 2013, **7**: 261. doi:10.3389/fnins.2013.00261.
- Moll J, Schulkin J: **Social attachment and aversion in human moral cognition.***Neurosci Biobehav Rev* 2009, **33**(3):456–465. doi:10.1016/j.neubiorev.2008.12.001.
- Moll J, De Oliveira-Souza R, Zahn R: **The neural basis of moral cognition: sentiments, concepts, and values.***Ann N Y Acad Sci* 2008, **1124**:161–180. doi:10.1196/annals.1440.005.
- Moll J et al.: **The moral affiliations of disgust: a functional MRI study.***Cogn Behav Neurol* 2005, **18**(1):68–78. doi:10.1097/01.wnn.0000152236.46475.a7.
- Moll J, de Oliveira-Souza R: **Moral judgments, emotions and the utilitarian brain.***Trends Cogn Sci* 2007, **11**(8):319–321. doi:10.1016/j.tics.2007.06.001.
- Moll J, de Oliveira-Souza R, Eslinger PJ: **Morals and the human brain: a working model.***Neuroreport* 2003, **14**(3):299–305. doi:10.1097/00001756-200303030-00001.
- Moll J, Schulkin J. **Social attachment and aversion in human moral cognition.***Neurosci Biobehav Rev* 2009, **33**(3):456–465. doi:10.1016/j.neubiorev.2008.12.001.
- Moll J, De Oliveira-Souza R: **When morality is hard to like.***Sci Am Mind* 2008, **19**(1):30–35. doi:10.1038/scientificamericanmind0208-30.
- Moll J, de Oliveira-Souza R, Bramati IE, Grafman J: **Functional networks in emotional moral and nonmoral social judgments.***Neuroimage* 2002, **16**(3):696–703. doi:10.1006/nimg.2002.1118.
- Moll J et al.: **The neural correlates of moral sensitivity: a functional magnetic resonance imaging investigation of basic and moral emotions.***J Neurosci* 2002, **22**(7):2730–2736. doi:10.1001/archpsyc.64.2.179.
- Moll J et al.: **Opinion: the neural basis of human moral cognition.***Nat Rev Neurosci* 2005, **6**(10):799–809. doi:10.1038/nrn1768.
- Moll J et al.: **The self as a moral agent: link-ling the neural bases of social agency and moral sensitivity.***Soc Neurosci* 2007, **2**(3–4):336–352. doi:10.1080/17470910701392024.
- Moran JM et al.: **Impaired theory of mind for moral judgment in high-functioning autism.***Proc Natl Acad Sci USA* 2011, **108**(7):2688–2692. doi:10.1073/pnas.1011734108.
- Moreno A, Etxeberria A, Umerez J: **The autonomy of biological individuals and artificial models.***Biosystems* 2008, **91**(2):309–319. doi:10.1016/j.biosystems.2007.05.009.
- Moreno J: **Society and the reception of imaging technology: the American experience.*** Cortex* 2011, **47**(10):1256–1258. doi:10.1016/j.cortex.2011.04.024.
- Moretto G, Làdavas E, Mattioli F, di Pellegrino G: **A psychophysiological investigation of moral judgment after ventromedial prefrontal damage.***J Cogn Neurosci* 2010, **22**(8):1888–1899. doi:10.1162/jocn.2009.21367.
- Morris SG: **Neuroscience and the free will conundrum.***Am J Bioeth* 2007, **7**(5):20–22. doi:10.1080/15265160701290298.
- Morrison NK, Severino SK: **Toward a biological framework for morality.***NeuroQuantology* 2005, **3**(4). doi:10.14704/nq.2005.3.4.78.
- Moya CJ: **Libertad, responsabilidad moral y neurociencia.***Pasajes: Revista de Pensamiento Contemporaneo* 2012, **37**:109–118.
- Mulert C et al.: E**vidence for a close relationship between conscious effort and anterior cingulate cortex activity.***Int J Psychophysiol* 2005, **56**(1):65–80. doi:10.1016/j.ijpsycho.2004.10.002.
- Müller S, Walter H: **Review autonomy: Implications of the neurosciences and the free will debate for the principle of respect for the patient’s autonomy.***Camb Q Healthc Ethics* 2010, **19**(2):205–217. doi:10.1017/s0963180109990478.
- Murphy FC et al.: **Assessing the automaticity of moral processing: efficient coding of moral information during narrative comprehension.***QJ Exp Psychol (Hove)* 2009, **62**(1):41–49. doi:10.1080/17470210802254441.
- Murphy N: **Whatever happened to the soul? Theological perspectives on neuroscience and the self.***Ann NY Acad Sci* 2003, **1001**:51–64. doi:10.1196/annals.1279.004.
- Myyry L, Helkama K: **Socio-cognitive conflict, emotions, and complexity of thought in real-life morality.***Scand J Psychol* 2007, **48**(3):247–259. doi:10.1111/j.1467-9450.2007.00579.x.
- Nahab FB et al.: **The neural processes underlying self-agency.***Cereb Cortex* 2011, **21**(1):48–55. doi:10.1093/cercor/bhq059.
- Nahra C: **Neuroscience of ethics: the state of art and the promises for the future.***Ethic@Revista Internacional de Filosofia da Moral* 2011, **10**(1):109–132. doi:10.5007/1677-2954.2011v10n1p109.
- Nakao H, Itakura S: **An integrated view of empathy: psychology, philosophy, and neuroscience.***Integr Psychol Behav Sci* 2009, **43**(1):42–52. doi:10.1007/s12124-008-9066-7.
- Nakao T, Ohira H, Northoff G: **Distinction between externally vs. internally guided decision-making: operational differences, meta-analytical comparisons and their theoretical implications.***Front Neurosci* 2012, **6**:31. doi:10.3389/fnins.2012.00031.
- Narvaez D: **Triune ethics: the neurobiological roots of our multiple moralities.***New Ideas Psychol* 2008, **26**(1):95–119.
- Narvaez D, Vaydich JL: **Moral development and behaviour under the spotlight of the neurobiological sciences.***J Moral Educ* 2008, **37**(3):289–312. doi:10.1080/03057240802227478.
- Nau JY:**Submissive mind exploration of ethical questions.***Rev Med Suisse* 2008, **4**(152):935.
- Nicolle A, Bach DR, Frith C, Dolan RJ: **Amygdala involvement in self-blame regret.***Soc Neurosci* 2011, **6**(2):178–189. doi:10.1080/17470919.2010.506128.
- Northoff G: **Neuroscience of decision making and informed consent: an investigation in neuroethics.***J Med Ethics* 2006, **32**(2):70–73. doi:10.1136/jme.2005.011858.
- Northoff G, Boeker H, Bogerts B: **Subjective experience and neuronal integration in the brain: do we need a first-person neuroscience?***Fortschr Neurol Psychiatr* 2006, **74**(11):627–634. doi:10.1055/s-2005-915610.
- Overman W et al.: **Contemplation of moral dilemmas eliminates sex differences on the Iowa gambling task.***Behav Neurosci* 2006, **120**(4):817–825. doi:10.1037/0735-7044.120.4.817.
- Parkinson C et al.: **Is morality unified? Evidence that distinct neural systems underlie moral judgments of harm, dishonesty, and disgust.***J Cogn Neurosci* 2011, **23**(10):3162–3180. doi:10.1162/jocn_a_00017.
- Pascual L, Rodrigues P, Gallardo-Pujol D: **How does morality work in the brain? A functional and structural perspective of moral behavior.***Front Integr Neurosci* 2013, **7**:65. doi:10.3389/fnint.2013.00065.
- Passos-Ferreira C: **Seria a moralidade determinada pelo cerebro? Neuronios-espelhos, empatia e neuromoralidade. [Does the brain have anything to do with morality? Mirror neurons, empathy and neuromorality].***Physis* 2011, **21**(2):471–490.
- Pastötter B, Gleixner S, Neuhauser T, Bäuml KH: **To push or not to push? Affective influences on moral judgment depend on decision frame.***Cognition* 2013, **126**(3):373–377. doi:10.1016/j.cognition.2012.11.003.
- Patterson R, Rothstein J, Barbey AK: **Reasoning, cognitive control, and moral intuition.***Front Integr Neurosci* 2012, **6**:114. doi:10.3389/fnint.2012.00114.
- Pfaff DW, Kavaliers M, Choleris E**: Mechanisms underlying an ability to behave ethically.***Am J Bioeth* 2008, **8**(5):10–19. doi:10.1080/15265160802179994.
- Phelps EA: **The neuroscience of a person network.***Am J Bioeth* 2007, **7**(1):49–50. doi:10.1080/15265160601064223.
- Pockett S: **The concept of free will: philosophy, neuroscience and the law.***Behav Sci Law* 2007, **25**(2):281–293. doi:10.1002/bsl.743.
- Powell R**: The biomedical enhancement of moral status.***J Med Ethics* 2013, **39**(2):65–66. doi:10.1136/medethics-2012-101312.
- Prehn K et al.: **Individual differences in moral judgment competence influence neural correlates of socio-normative judgments.*** Soc Cogn Affect Neurosci* 2008, **3**(1):33–46. doi:10.1093/scan/nsm037.
- Preston JL, Ritter RS, Hepler J: **Neuroscience and the soul: competing explanations for the human experience.***Cognition* 2013, **127**(1):31–37. doi:10.1016/j.cognition.2012.12.003.
- Price JL: **Free will versus survival: brain systems that underlie intrinsic constraints on behavior.***J Comp Neurol* 2005, **493**(1):132–139. doi:10.1002/cne.20750.
- Prinz J: **The emotional basis of moral judgments.***Philos Explor* 2006, **9**(1):29–43. doi:10.1080/13869790500492466.
- Pyysiäinen I, Hauser M: **The origins of religion: evolved adaptation or by-product*?****Trends Cogn Sci* 2010, **14**(3):104–109. doi:10.1016/j.tics.2009.12.007.
- Radman Z: **The ethical mind: an outline.***Synth Philos* 2006, **21**(2):385–394.
- Raine A, Yang Y: **Neural foundations to moral reasoning and antisocial behavior.***Soc Cogn Affect Neurosci* 2006, **1**(3):2-3-213. doi:10.1093/scan/nsl03.3.
- Rapley M, McCarthy D, McHoul A: **Mentality or morality? Membership categorization, multiple meanings and mass murder.***Br J Soc Psychol* 2003, **42**(3):427–444. doi:10.1348/014466603322438242.
- Rasmusson A: **Neuroethics as a brain-based philosophy of life: the case of Michael S. Gazzaniga.***Neuroethics* 2009, **2**(1):3–11. doi:10.1007/s12152-008-9024-6.
- Ravven HM: **Spinoza’s anticipation of contemporary affective neuroscience.***Consciousness & Emotion* 2003, **4**(2):257–290.
- Reichlin M: **The challenges of neuroethics.***Funct Neurol* 2007, **22**(4):219–234.
- Rengifo FJ: **“La responsabilidad del sujeto”.***Desde el Jardin de Freud* 2005, **5**:144–155.
- Reniers RL et al.: **Moral decision-making, ToM, empathy and the default mode network.***Biol Psychol* 2012, **90**(3):202–210. doi:10.1016/j.biopsycho.2012.03.009.
- Reynolds SJ: **A neurocognitive model of the ethical decision-making process: implications for study and practice.***J Appl Psychol* 2006, **91**(4):737–748. doi:10.1037/0021-9010.91.4.737.
- Rilling JK et al.: **A neural basis for social cooperation.***Neuron* 2002, **35**(2):395–405. doi:10.1016/S0896-6273(02)00755-9.
- Robertson D et al.: **The neural processing of moral sensitivity to issues of justice and care.***Neuropsychologia* 2007, **45**(4):755–766. doi:10.1016/j.neuropsychologia.2006.08.014.
- Robinson DN: **Big brains and Boskops: phrenology lives!***Theory Psychol* 2013, **23**(3):391–396. doi:10.1177/0959354312471741.
- Rockwell T: **Where should we look for mental representations? On the need for epistemic ethics.***Account Res* 2013, **20**(1):42–56. doi:10.1080/08989621.2013.749746.
- Rohde M, Stewart J: **Ascriptional and ‘genuine’ autonomy.***Biosystems* 2008, **91**(2):424–433. doi:10.1016/j.biosystems.2007.05.017.
- Rose N: **Screen and intervene: governing risky brains.***Hist Human Sci* 2010, **23**(1):79–105. doi:10.1177/0952695109352415.
- Roskies AL: **Neuroscientific challenges to free will and responsibility.***Trends Cogn Sci* 2006, **10**(9):419–423. doi:10.1016/j.tics.2006.07.011.
- Roskies AL: **How does the neuroscience of decision making bear on our understanding of moral responsibility and free will?***Curr Opin Neurobiol* 2012, **22**(6):1022–1026. doi:10.1016/j.conb.2012.05.009.
- Roskies, AL: **Response to Sie and Wouters: a neuroscientific challenge to free will and responsibility?***Trends Cogn Sci* 2008, **12**(1):4. doi:10.1016/j.tics.2007.10.003.
- Roskies AL: **A puzzle about empathy.***Emot Rev* 2011, **3**(3):278–280. doi:10.1177/1754073911402395.
- Roskies A: **Are ethical judgments intrinsically motivational? Lessons from “acquired sociopathy”.***Philos Psychol* 2003, **16**(1):51–66. doi:10.1080/0951508032000067743.
- Rubia-Vila FJ. **The ghost of the free will.***An R Acad Nac Med (Madr*) 2009, **126**(3):377–387.
- Sandberg J, Juth N: **Ethics and intuitions: a reply to Singer.***J Ethics* 2011, **15**(3):209–226. doi:10.1007/s10892-010-9088-5.
- Sarlo M et al.: **Temporal dynamics of cognitive-emotional interplay in moral decision-making.***J Cogn Neurosci* 2012, **24**(4):1018–1029. doi:10.1162/jocn_a_00146.
- Schaefer GO: **What is the goal of moral engineering**? *AJOB Neurosci* 2011, **2**(4):10–11.doi:10.1080/21507740.2011.620593.
- Schleim S, Schirmann F: **Philosophical implications and multidisciplinary challenges of moral physiology.***TRAMES-J Humanit Soc* 2011, **15**(2):127–146. doi:10.3176/tr.2011.2.02.
- Schleim S: **Moral physiology, its limitations and philosophical implications.***Jahr Wiss Ethik* 2008, **13**:51–80.
- Schirmann F: **Invoking the brain in studying morality: a theoretical and historical perspective on the neuroscience of morality.***Theory Psychol* 2013, **23**(3):289–304. doi:10.1177/0959354313478479.
- Schoenherr-Mann HM: **Willensfreiheit und Verantwortung zwischen Philosophie und Hirnforschung [Free will and responsibility between philosophy and brain research].***Ethica* 2012, **20**(3):237–253.
- Seok B: **Neuroscience, moral sentimentalism, and Confucian philosophy: moral psychology of the body and emotion.***APA Newsl: Asian Phil* 2013, **13**(1):2–9.
- Severino SK: **Free will according to John Duns Scotus and neuroscience.***Zygon* 2012, **47**(1):156–174. doi:10.1111/j.1467-9744.2011.01244.x.
- Shadlen MN, Roskies AL: **The neurobiology of decision-making and responsibility: reconciling mechanism and mindedness.***Front Neurosci* 2012, **6**:56. doi:10.3389/fnins.2012.00056.
- Shalvi S, Eldar O, Bereby-Meyer Y: **Honesty requires time (and lack of justifications).***Psychol Sci* 2012, **23**(10):1264–1270. doi:10.1177/0956797612443835.
- Shenhav A, Greene JD: **Moral judgments recruit domain-general valuations mechanisms to integrate representations of probability and magnitude.***Neuron* 2010, **67**(4):667–677. doi:10.1016/j.neuron.2010.07.020.
- Shermer M: **The science of right and wrong. Can data determine moral values?***Sci Am* 2011, **304**(1):83.
- Shoemaker WJ: **The social brain network and human moral behavior.***Zygon* 2012, **47**(4):806–820. doi:10.1111/j.1467-9744.2012.01295.x.
- Sie M, Wouters A: **The BCN challenge to compatibilist free will and personal responsibility.***Neuroethics* 2010, **3**(2):121–133. doi:10.1007/s12152-009-9054-8.
- Sie M, Wouters A: **The real challenge to free will and responsibility.***Trends Cogn Sci* 2008, **12**(1):3–4. doi:10.1016.j.tics.2007.10.005.
- Sie M: **Moral agency, conscious control, and deliberative awareness.***Inquiry***52**(5):516–531. doi:10.1080/00201740903302642
- Siegal M, Varley R: **Neural systems involved in “theory of mind.”***Nat Rev Neurosci* 2002, **3**(6):463–471. doi:10.1038/nrn844.
- Singer P: **Ethics and intuitions.***J Ethics* 2005, **9**(3–4):331–352. doi:10.1007/s10892-005-3508-y.
- Singer T et al.: **Empathic neural responses are modulated by the perceived fairness of others.***Nature* 2006, **439**(7075):466–469. doi:10.1038/nature04271.
- Singer T et al.: **Brain responses to the acquired moral status of faces.***Neuron* 2004, **41**(4):653–662. doi:10.1016/s0896-6273(04)00014-5.
- Singer T: **The neuronal basis of empathy and fairness.***Novartis Found Symp* 2007, **278**:20–30.
- Sinnott-Armstrong W: **Does morality have an essence?***Psychol Inq* 2012, **23**(2):194–197. doi:10.1080/1047840x.2012.666653.
- Sinnott-Armstrong W, Wheatley T: **The disunity of morality and why it matters to philosophy.***The Monist* 2012, **95**(3):355–377. doi:10.5840/monist201295319.
- Slachevsky A, Silva JR, Prenafeta ML, Novoa F: **The contribution of neuroscience to the understanding of moral behavior.***Rev Med Chil* 2009, **137**(3):419–425. doi:/S0034-98872009000300015.
- Slingerland E: **“Of what use are the odes?” cognitive science, virtue ethics, and early Confucian ethics.***Philos East West* 2011, **61**(1):80–109.
- Smetana JG, Killen M: **Moral cognition, emotions, and neuroscience: An integrative developmental view.***International Journal of Developmental Science* 2008, **2**(3):324–339. doi:10.3233/DEV-2008-2309.
- Smith K: **Neuroscience vs. philosophy: taking aim at free will.***Nature* 2011, **477**(7362):23–25. doi:10.1038/477023a.
- Smith MJ: **Conceptualizing the “self” in neuroethics: an appeal to philosophy of mind.***AJOB Neurosci* 2010, **1**(3):16–17. doi:10.1080/21507740.2010.485271.
- Sokol BW, Chandler MJ, Jones C: **From mechanical to autonomous agency: the relationship between children’s moral judgments and their developing theories of mind.***New Dir Child Adolesc Dev* 2004, **103**:19–36. doi:10.1002/cd.95.
- Sommer M et al.: **How should I decide? the neural correlates of everyday moral reasoning.***Neuropsychologia* 2010, **48**(7):2018–2026. doi:10.1016/j.neuropsychologia.2010.03.023.
- Sousa P, Holbrook C, Piazza J: **The morality of harm.***Cognition* 2009, **113**(1):80–92. doi:10.1016/j.cognition.2009.06.015.
- Spezio M: **The neuroscience of emotion and reasoning in social contexts: implications for moral theology.***Mod Theol* 2011, **27**(2):339–356. doi:10.1111/j.1468-0025.2010.01680.x.
- Suhler CL, Churchland P: **Can innate, modular “foundations” explain morality? challenges for Haidt's moral foundations theory.***J Cogn Neurosci* 2011, **23**(9):2103–2116. doi:10.1162/jocn.2011.21637.
- Tachiki N: **A natural science approach to the study of morality: recent developments in the study of morality through the cooperation between cognitive neuroscience and evolutionary biology.***Studies in Moralogy: The Forum for Ethical and Moral Studies*, 2013, **71**:39–49.
- Takala T, Buller T: **Neural grafting: implications for personal identity and personality.***TRAMES-J Humanit Soc*, 2011, **15**(2):168–178. doi:10.3176/tr.2011.2.05.T.
- Talmi D, Frith C: **Neurobiology: feeling right about doing right.***Nature* 2007, **446**(7138):865–866. doi:10.1038/446865a.
- Tancredi LR: **The neuroscience of “free will”.***Behav Sci Law* 2007, **25**(2):295–308. doi:10.1002/bsl.749.
- Tankersley D: **Psychopathology, neuroscience, and moral theory.***Philos Psychiatr Psychol* 2011, **18**(4):349–357. doi:10.1353/ppp.2011.0044.
- Tankersley D: **Neuromorality.***Philos Psychiatr Psychol* 2011, **18**(4):367–369. doi:10.1353/ppp.2011.0053.
- Tassy S: **On the necessity to distinguishing judgment from subjective choice in the cognitive neuroscience of morality].***Med Sci (Paris)* 2011, **27**(10):889–894. doi:10.1051/medsci/20112710018.
- Tassy S, Le Coz P, Wicker B: **Current knowledge in moral cognition can improve medical ethics.***J Med Ethics* 2008, **34**(9):679–682. doi:10.1136/jme.2006.018812.
- Taylor PL: **Responsibility rewarded: ethics, engagement, and scientific autonomy in the labyrinth of the minotaur.***Neuron* 2011, **70**(4):577–581. doi:10.1016/j.neuron.2011.05.009.
- Tersman F: **The reliability of moral intuitions: a challenge from neuroscience.***Australas J Philos* 2008, **86**(3):389–405. doi:10.1080/00048400802002010.
- Thagard P: **The moral psychology of conflicts of interest: insights from affective neuroscience.***J Appl Philos* 2007, **24**(4):367–380. doi:10.1111/j.1468-5930.2007.00382.x.
- Thomas BC, Croft KE, Tranel D: **Harming kin to save strangers: further evidence for abnormally utilitarian moral judgments after ventromedial prefrontal damage.***J Cogn Neurosci* 2011, **23**(9):2186–2196. doi:10.1162/jocn.2010.21591.
- Trémolière B, Neys WD, Bonnefon JF: **Mortality salience and morality: thinking about death makes people less utilitarian.***Cognition* 2012, **124**(3):379–384. doi:10.1016/j.cognition.2012.05.011.
- Tsomo KL: **Compassion, ethics and neuroscience: neuroethics through Buddhist eyes.***Sci Eng Ethics* 2012, **18**(3):529–537. doi:10.1007/s11948-012-9369-4.
- Tsukiura T, Cabeza R: **Shared brain activity for aesthetic and moral judgments: implications for the Beauty-is-Good stereotype.***Soc Cogn Affect Neurosci* 2011, **6**(1):138–148. doi:10.1093/scan/nsq025.
- Tucker P: **Reinventing morality: evolutionary biology and neuroscience are adding to our understanding of a historically unscientific area.***Futurist* 2009, **43**(1):20.
- Van Berkum JJ et al.: **Right or wrong? The brain’s fast response to morally objectionable statements.***Psychol Sci* 2009, **20**(9):1092–1099. doi:10.1111/j.1467-9280.2009.02411.x.
- Velez Garcia AE, Ostrosky-Solis F: **From morality to moral emotions.***Int J Psychol* 2006, **41**(5):348–354. doi:10.1080/00207590500345898.
- von Lautz A, Maier S: **Autonomy, decisions, and free will.***Cogn Process* 2011, **12**(3):311–313. doi:10.1007/s10339-011-0409-9.
- Wain O, Spinella M: **Executive functions in morality, religion, and paranormal beliefs.***Int J Neurosci* 2007, **117**(1):135–146. doi:10.1080/00207450500534068.
- Wainryb C: **“Is” and “ought”: moral judgments about the world as understood.***New Dir Child Adolesc Dev* 2004, **103**:3–18. doi:10.1002/cd.94.
- Waller RR: **Beyond button presses: the neuroscience of free and morally appraisable actions.***Monist* 2012, **95**(3):441–462. doi:10.5840/monist201295323.
- Walter H: **Emotional dysfunction, psychopathy, and cognitive neuroscience. What is new and what are the consequences.***Nervenarzt* 2005, **76**(5):557–568.
- Woodward J, Allman J: **Moral intuition: its neural substrates and normative significance.***J Physiol Paris* 2007, **101**(4–6):179–202. doi:10.1016/j.jphysparis.2007.12.003.
- Wu Y et al.: **Brain activity in fairness consideration during asset distribution: does the initial ownership play a role?***PLoS One* 2012, **7**(6):e39627. doi:10.1371/journal.pone.0039627.
- Xue SW, Wang Y, Tang YY: **Personal and impersonal stimuli differentially engage brain networks during moral reasoning.***Brain Cogn* 2013, **81**(1):24–28. doi:10.1016/j.bandc.2012.09.004.
- Young L, Koenigs M: **Investigating emotion in moral cognition: a review of evidence from functional neuroimaging and neuropsychology.***Br Med Bull* 2007, **84**:69–79. doi:10.1093/bmb/ldm031.
- Young L et al.: **Disruption of the right temporoparietal junction with transcranial magnetic stimulation reduces the role of beliefs in moral judgments.***Proc Natl Acad Sci USA* 2010, **107**(15):6753–6758. doi:10.1073/pnas.0914826107.
- Young L, Cushman F, Hauser M, Saxe R: **The neural basis of the interaction between theory of mind and moral judgment.***Proc Natl Acad Sci USA* 2007, **104**(20):8235–8240. doi:10.1073/pnas.0701408104.
- Young L, Dungan J: **Where in the brain is morality? Everywhere and maybe nowhere.***Soc Neurosci* 2012, **7**(1):1–10. doi:10.1080/17470919.2011.569146.
- Young L, Saxe R: **An fMRI investigation of spontaneous mental state inference for moral judgment.***J Cogn Neurosci* 2009, **21**(7):1396–1405. doi:10.1162/jocn.2009.21137.
- Young L, Saxe R: **It’s not just what you do, but what’s on your mind: a review of Kwame Anthony Appiah’s “Experiments in Ethics”.***Neuroethics* 2010, **3**(3): 201–207. doi:10.1007/s12152-010-9066-4.
- Young L, Saxe R: **The neural basis of belief encoding and integration in moral judgment.***Neuroimage* 2008, **40**(4):1912–1920. doi:10.1016/j.neuroimage.2008.01.057.
- Youssef FF et al.: **Stress alters personal moral decision making.***Psychoneuroendocrinology* 2012, **37**(4):491–498. doi:10.1016/j.psyneuen.2011.07.017.
- Zaidel DW, Nadal M: **Brain intersections of aesthetics and morals: perspectives from biology, neuroscience, and evolution.***Perspect Bio Med* 2011, **54**(3):367–380. doi:10.1353/pbm.2011.0032.
- Zak PJ, Kurzban R, Matzner WT: **The neurobiology of trust.***Ann NY Acad Sci* 2004, **1032**:224–227. doi:10.1196/annals.1314.025.
- Zak PJ, Kurzban R, Matzner WT: **Oxytocin is associated with human trustworthiness.***Horm Behav* 2005, **48**(5):522–527. doi:10.1016/j.yhbeh.2005.07.009.
- Zak PJ, Fakhar A: **Neuroactive hormones and interpersonal trust: international evidence.***Econ Hum Biol* 2006, **4**(3):412–429. doi:10.1016/j.ehb.2006.06.004.
- Zak PJ: **The neurobiology of trust.***Sci Am* 2008, **298**(6):88–95. doi:10.1038/scientificamerican0608-88.
- Zak PJ, Barraza JA: **The neurobiology of collective action.***Front Neurosci* 2013, **7**:211. doi:10.3389/fnins.2013.00211.
- Zarpentine, C: **Neuroethics and the complexity of moral psychology.***AJOB Neurosci* 2011, **2**(2):12–13. doi:10.1080/21507740.2011.559920.
- Zhang L: **Moral judgment under perspective of neuroethics.***Zi ran bian zheng fa tong xun = Journal of Dialectics of Nature* 2008, **3**:15.

### Books

- Baertschi B: *La Neuroéthique: Ce que les Neurosciences font à Nos Conceptions Morales.* Paris: Découverte; 2009.
- Baron-Cohen S, Lombardo M, Tager-Flusberg H: (Eds): *Understanding Other Minds: Perspectives from Developmental Social Neuroscience.* Oxford, U.K.: Oxford University Press; 2013.
- Bickle J: (Ed)*: The Oxford Handbook of Philosophy of Neuroscience.* New York: Oxford University Press; 2009.
- Bickle J: *Philosophy and Neuroscience: A Ruthlessly Reductive Account.* Amsterdam: Kluwer; 2003.
- Boella L: *Neuroetica: La Morale Prima della Morale.* Milano: Raffaello Cortina; 2008.
- Brugs R, Issa AM, Postiglione M: *Advances in Neuroscience: Social, Moral, Philosophical, Theological Implications: Proceedings of the ITEST Workshop, September, 2002.* St. Louis, Mo.: ITEST Faith/Science Press; 2003.
- Burton RA: *A Skeptic's Guide to the Mind: What Neuroscience Can and Cannot Tell Us about Ourselves.* New York: St. Martin's Press; 2013.
- Cacioppo JT et al.: (Eds): *Foundations in Social Neuroscience.* Cambridge, Mass.: MIT Press; 2002.
- Callender JS: *Free Will and Responsibility: A Guide for Practitioners.* Oxford, U.K.: Oxford University Press; 2010.
- Casebeer WD: *Natural Ethical Facts: Evolution, Connectionism, and Moral Cognition.* Cambridge, Mass.: MIT Press; 2003.
- Changeux JP: (Ed): *Neurobiology of Human Values.* Berlin: Springer; 2005.
- Churchland PM: *Neurophilosophy at Work.* Cambridge: Cambridge University Press; 2007.
- Churchland PS: *Braintrust: What Neuroscience Tells Us about Morality.* Princeton: Princeton University Press; 2011.
- Churchland PS*: Brain-Wise: Studies in Neurophilosophy.* Cambridge, Mass.: MIT Press; 2002.
- Clausen J, Maier G, Müller O: *Die 'Natur des Menschen' in Neurowissenschaft und Neuroethik.* Würzburg, Germany: Königshausen & Neumann; 2007.
- Clayton P, Schloss J: (Eds): *Evolution and Ethics*: *Human Morality in Biological and Religious Perspective.* Grand Rapids, Mich.: W. B. Eerdmans Publishing Company; 2004.
- Decety J, Ickes W: (Eds): *The Social Neuroscience of Empathy.* Cambridge, Mass.: MIT Press; 2009.
- DiSilvestro R: *Human Capacities and Moral Status.* Dordrecht: Springer; 2010.
- Ebstein R, Shamay-Tsoory S, Chew SH: (Eds): *From DNA to Social Cognition.* Hoboken, N.J.: Wiley-Blackwell; 2011.
- Engels EM, Hildt E: *Neurowissenschaften und Menschenbild.* Paderborn: Mentis; 2005.
- Franks DD: *Neurosociology: The Nexus between Neuroscience and Social Psychology.* New York: Springer; 2010.
- Gallagher S: *How the Body Shapes the Mind.* Oxford, U.K.: Clarendon Press; 2005.
- Gazzaniga, MS: *The Ethical Brain: The Science of Our Moral Dilemmas.* New York: Harper Perennial; 2005.
- Gazzaniga, MS: *Wann ist der Mensch ein Mensch? Antworten der Neurowissenschaft auf ethische Fragen.* Düsseldorf: Patmos-Verl; 2007.
- Gazzaniga MS: *El Cerebro Ético.* Barcelona: Paidós; 2006.
- Geyer C: *Hirnforschung und Willensfreiheit: zur Deutung der neuesten Experimente.* Frankfurt: Suhrkamp; 2004.
- Gillett G: *Subjectivity and Being Somebody: Human Identity and Neuroethics.* Exeter, UK: Imprint Academic; 2008.
- Giménez Amaya JM, Sánchez-Migallón Granados S*. De la Neurociencia a la Neurología: Narrativa Científica y Reflexión Filosófica.* Pamplona: Eunsa; 2010.
- Glimcher PW: *Decisions, Uncertainty, and the Brain: The Science of Neuroeconomics.* Cambridge, Mass.: MIT Press; 2003.
- Gluchman V: (Ed): *Morality: Reasoning on Different Approaches.* Amsterdam: Rodopi, 2013.
- Greene JD: *Moral Tribes: Emotion, Reason, and the Gap Between Us and Them.* New York: The Penguin Press; 2013.
- Hagner M: *Geniale Gehirne: Zur Geschichte der Elitegehirnforschung.* Göttingen: Wallstein Verlag; 2004.
- Harris S: *The Moral Landscape: How Science Can Determine Human Values.* New York: Free Press; 2010.
- Hauser MD: *Moral Minds: How Nature Designed Our Universal Sense of Right and Wrong.* New York: Ecco; 2006.
- Heintel P, Broer K: *Hirnforschung als dialektische Sozialwissenschaft.* Münster: Wien Lit; 2005.
- Jackson F, Pettit P, Smith M: *Mind, Morality, and Explanation: Selected Collaborations.* Oxford, U.K.: Oxford University Press; 2004.
- Legrenzi P, Umiltà C: *Neuromania: On the Limits of Brain Science.* Translated by Frances Anderson. Oxford, U.K.: Oxford University Press; 2011.
- Lehrer J: *How We Decide.* Boston: Houghton Mifflin Harcourt; 2009.
- Metzinger T: *The Ego Tunnel: The Science of the Mind and the Myth of the Self.* New York: Basic Books; 2009.
- Murphy NC, Ellis GFR, O’Connor T: *Downward Causation and the Neurobiology of Free Will.* Berlin: Springer Verlag; 2009.
- Noë A: *Out of Our Heads: Why You Are Not Your Brain, and Other Lessons from the Biology of Consciousness.* New York: Hill and Wang; 2009.
- Pfaff DW: *The Neuroscience of Fair Play: Why We (Usually) Follow the Golden Rule.* New York: Dana Press; 2007.
- Prinz JJ. T*he Emotional Construction of Morals.* Oxford, U.K.: Oxford University Press; 2007.
- Quartz SR, Sejnowski TJ: *Liars, Lovers and Heroes: What the New Brain Science Reveals About How We Become Who We Are.* New York: William Morrow; 2002.
- Rees DA, Rose SPR: *The New Brain Sciences: Perils and Prospects.* New York: Cambridge University Press; 2004.
- Reuter-Lorenz PA, Baynes K, Mangun GR, Phelps EA: (Eds)*. The Cognitive Neuroscience of Mind: A Tribute to Michael S. Gazzaniga.* Cambridge, Mass.: MIT Press, 2010.
- Rolls ET*: Memory, Attention, and Decision-Making: A Unifying Computational Neuroscience Approach.* Oxford, U.K.: Oxford University Press; 2008.
- Scharifi G: Brauchen *Brauchen wir eine neue Ethik?: Herausforderungen der Ethik durch die Neurowissenschaft.* Paderborn: Mentis; 2011.
- Schroeder P, Roskies AL, Nichols S: *Moral Motivation.* Oxford, U.K.: Oxford University Press; 2010.
- Severino SK: *Behold Our Moral Body. Psychiatry, Duns Scotus, and Neuroscience.* Berlin: De Gruyter; 2013.
- Sinnott-Armstrong W: (Ed): *Moral Psychology, Volume 1: The Evolution of Morality—Adaptations and Innateness.* Cambridge, Mass.: MIT Press; 2007.
- Sinnott-Armstrong W: (Ed): *Moral Psychology, Volume 2: The Cognitive Science of Morality: Intuition and Diversity.* Cambridge, Mass.: MIT Press; 2008.
- Sinnott-Armstrong W: (Ed): *Moral Psychology, Volume 3: The Neuroscience of Morality: Emotion, Brain Disorders, and Development.* Cambridge, Mass.: MIT Press; 2008.
- Smith R: *Free Will and the Human Sciences in Britain, 1870–1910.* London: Pickering & Chatto; 2013.
- Sternberg EJ: *My Brain Made Me Do It: The Rise of Neuroscience and the Threat to Moral Responsibility.* Amherst, N.Y.: Prometheus Books; 2010.
- Szasz TS: *The Meaning of Mind: Language, Morality, and Neuroscience.* Syracuse, N.Y.: Syracuse University Press; 2002.
- Tancredi LR: *Hardwired Behavior: What Neuroscience Reveals about Morality.* Cambridge: Cambridge University Press; 2005.
- Taylor KE: *The Brain Supremacy: Notes from the Frontiers of Neuroscience.* Oxford, U.K.: Oxford University Press; 2012.
- Thiele LP*: The Heart of Judgment: Practical Wisdom, Neuroscience, and Narrative.* New York: Cambridge University Press; 2006.
- Thompson E: *Mind in Life: Biology, Phenomenology, and the Sciences of Mind.* Cambridge, Mass.: Belknap Press; 2007.
- Tracy JL, Robins RW, Tangney JP: (Eds): *The Self-Conscious Emotions: Theory and Research.* New York: Guilford Press; 2007.
- Verplaetse J, De Schrijver J, Vanneste S, Braeckman, J: (Eds): *The Moral Brain: Essays on the Evolutionary and Neuroscientific Aspects of Morality.* New York: Springer; 2009.
- Verplaetse J: *Localizing the Moral Sense: Neuroscience and the Search for the Cerebral Seat of Morality, 1800–1930.* Dordrecht: Springer; 2009.
- Verplaetse J: *Het Morele Brein: Een Geschiedenis Over de Plaats van de Moraal in Onze Hersenen.* Antwerp: Garant; 2006.
- Vilarroya O, i Argimon FF: (Eds): *Social Brain Matters: Stances on the Neurobiology of Social Cognition.* Amsterdam: Rodopi; 2007.
- Volavka J: *Neurobiology of Violence.* Washington, DC: American Psychiatric Publishing; 2002.

### Book chapters

- Ackerman S: **Neuroscience and morality.** In her *Hard Science, Hard Choices: Facts, Ethics, and Policies Guiding Brain Science Today.* New York: Dana Press; 2006:17–20.
- Ackerman S: **Moral decision making in the brain.** In her *Hard Science, Hard Choices: Facts, Ethics, and Policies Guiding Brain Science Today.* New York: Dana Press; 2006:48–51.
- Appiah A: **The varieties of moral experience.** In his *Experiments in Ethics.* Boston: Harvard University Press; 2008:121–163.
- Au C: **Eine zwei-quellen-theorie der Moral: spekulative Gedanken vor dem hintergrund einer neurologischen Fallstudie.** In *Zeithorizonte des Ethischen: zur Bedeutung der Temporalität in der Fundamental- und Bioethik.* Edited by Georg Pfleiderer; Christoph Rehmann-Sutter (Hrsg.). [… Forschungssymposium “Zeithorizonte des Ethischen”, das wir im Landgut Castelen bei Kaiseraugust vom 16. bis 18. Oktober 2003 organisiert hatten]. Stuttgart: Kohlhammer; 2006:191–200.
- Blair RJ: **Contributions of neuroscience to the understanding of moral reasoning and its development.** In *Developmental Social Cognitive Neuroscience.* Edited by Philip D. Zelazo, Michael Chandler, Eveline Crone. New York: Psychology Press; 2010:269–288.
- Buller S: **Brains, lies, and psychological explanations.** In *Neuroethics: Defining the Issues in Theory, Practice, and Policy.* Edited by Judy Illes. Oxford, U.K.: Oxford University Press; 2006:51–60.
- Casebeer WD: **Moral cognition and its neural constituents.** In *Defining Right and Wrong in Brain Science: Essential Readings in Neuroethics.* Edited by Walter Glannon. New York: Dana Press; 2007:206–220.
- Churchland PM: **Toward a cognitive neurobiology of the moral virtues.** In *Scientific and Philosophical Perspectives in Neuroethics.* Edited by James Giordano, Bert Gordijn. Cambridge: Cambridge University Press; 2010:146–171.
- Churchland PS: **Neuroscience: reflections on the neural basis of morality.** In *Defining Right and Wrong in Brain Science: Essential Readings in Neuroethics.* Edited by Walter Glannon. New York: Dana Press; 2007:179–182.
- Churchland PS: **Moral decision making and the brain.** In *Neuroethics: Defining the Issues in Theory, Practice, and Policy.* Edited by Judy Illes. Oxford, U.K.: Oxford University Press; 2006:3–16.
- Churchland PS: **Inference to the best decision.** In *The Oxford Handbook of Philosophy and Neuroscience.* Edited by John Bickle. Oxford, U.K.: Oxford University Press; 2009:419–430.
- Churchland PS: **Human dignity from a neurophilosophical perspective.** In *Human Dignity and Bioethics: Essays Commissioned by the President's Council on Bioethics.* Washington, DC: President’s Council on Bioethics; 2008:99–121.
- Damasio A: **The neural basis of social behavior: ethical implications.** In *Defining Right and Wrong in Brain Science: Essential Readings in Neuroethics.* Edited by Walter Glannon. New York: Dana Press; 2007:175–178.
- Decety J, Howard LH: **A neurodevelopmental perspective on morality.** In *Handbook of Moral Development.* Edited by Melanie Killen, Judith G. Smetana. New York: Psychology Press; 2013:454–474.
- Decety J, Batson CD. **Empathy and morality: integrating social and neuroscience approaches.** In *The Moral Brain: Essays on the Evolutionary and Neuroscientific Aspects of Morality.* Edited by Jan Verplaetse, Jelle De Schrijver, Sven Vanneste, Johan Braeckman. Netherlands: Springer; 2009:109–127.
- Donald MW: **The definition of human nature.** In *The New Brain Sciences: Perils and Prospects.* Edited by Dai Rees, Steven Rose. New York: Cambridge University Press; 2004:34–58.
- Farah MJ, Heberlein AS: **Personhood: an illusion rooted in brain function?** In *Neuroethics: An Introduction with Readings.* Edited by Martha J. Farah. Cambridge, MA: MIT Press; 2010:321–338.
- Gazzaniga MS: **The brain produces a poor autobiography.** In his *The Ethical Brain: The Science of Our Moral Dilemmas.* New York: Harper Perennial; 2006:120–144.
- Gazzaniga M: **My brain made me do it.** In *Defining Right and Wrong in Brain Science: Essential Readings in Neuroethics.* Edited by Walter Glannon. New York: Dana Press; 2007: 183–194.
- Gillett G: **The moral subject.** In his *Subjectivity and Being Somebody: Human Identity and Neuroethics.* Charlottesville: Imprint Academic; 2008:84–106.
- Gillet G: **On method in moral science.** In his *Subjectivity and Being Somebody: Human Identity and Neuroethics.* Charlottesville: Imprint Academic; 2008:253–255.
- Glannon W: **Brain, body, and self.** In his *Bioethics and the Brain.* New York: Oxford University Press; 2007:13–44.
- Glannon W**: Our brains are not us.** In his *Brain, Body, and Mind: Neuroethics with a Human Face.* New York: Oxford University Press; 2011:11–40.
- Glannon W: **Neuroscience, free will, and moral responsibility.** 41(31); In his *Brain, Body, and Mind: Neuroethics with a Human Face.* New York: Oxford University Press; 2011:41–71.
- Glannon W: **Neuroscience and moral reasoning.** In his *Brain, Body, and Mind: Neuroethics with a Human Face.* New York: Oxford University Press; 2011:93–114.
- Greene J: **From neural ‘is’ to moral ‘ought’: what are the moral implications of neuroscientific moral psychology?** In *Defining Right and Wrong in Brain Science: Essential Readings in Neuroethics.* Edited by Walter Glannon. New York: Dana Press; 2007:221–236.
- Greene J: **Cognitive nuroscience and the structure of the moral mind.** In *The Innate Mind, Vol. 1.* Edited by Peter Carruthers, Stephen Laurence, Stephen Stich. New York: Oxford University Press; 2005:338–352.
- Greene JD: **The secret joke of Kant’s soul.** In *Moral Psychology, Vol. 3: The Neuroscience of Morality: Emotion, Disease, and Development.* Edited by W. Sinnott-Armstrong. Cambridge, Mass.: MIT Press; 2007:35–80.
- Greene JD: **The cognitive neuroscience of moral judgment.** In *The Cognitive Neurosciences.* Edited by Michael S. Gazzaniga et al. Cambridge, Mass.: MIT Press; 2009:987–1002.
- Haggard P: **Neuroethics of free will.** In *The Oxford Handbook of Neuroethics.* Edited by Judy Illes, Barbara J. Sahakian. Oxford, U.K.: Oxford University Press; 2013:219–226.
- Haidt J: **The moral emotions.** In *Handbook of Affective Sciences.* Edited by Richard J. Davidson, Klaus R. Scherer, and H. Hill Goldsmith. Oxford, U.K.: Oxford University Press; 2003:852–870.
- Joyce R: **What neuroscience can (and cannot) contribute to metaethics.** In *Moral Psychology, Vol. 3: The Neuroscience of Morality: Emotion, Disease, and Development.* Edited by W Sinnott-Armstrong. Singapore: Wiley-Blackwell; 2007:371–394.
- Koenigs M: **Emotion and moral cognition.** In *From DNA to Social Cognition.* Hoboken, N.J.: Wiley-Blackwell; 2012:111–122.
- Kollek R: **Mind metaphors, neurosciences and ethics.** In *The New Brain Sciences: Perils and Prospects.* Edited by Dai Rees, Steven Rose. New York: Cambridge University Press; 2004:71–87.
- Landreth A: **The emerging theory of motivation.** In *The Oxford Handbook of Philosophy and Neuroscience.* Edited by John Bickle. Oxford, U.K.: Oxford University Press; 2009:381–418.
- Levy N: **The neuroscience of free will.** In his *Neuroethics: Challenges for the 21st Century.* New York: Cambridge University Press; 2007:222–257.
- Meilaender G: **Commentary on Churchland.** In *Human Dignity and Bioethics: Essays Commissioned by the President's Council on Bioethics.* Washington, DC: President’s Council on Bioethics; 2008: 122–125.
- Mele A: **Free will and neuroscience.** In his *Free Will and Luck.* Oxford, U.K.: Oxford University Press, 2006:30–48.
- Midgley M: **Do we ever really act?** In *The New Brain Sciences: Perils and Prospects.* Edited by Dai Rees, Steven Rose. New York: Cambridge University Press; 2004:17–33.
- Moll J, de Oliveira-Souza R, Zahn R: **Neuroscience and morality: moral judgments, sentiments, and values.** In *Personality, Identity, and Character.* Edited by Darcia Narvaez, Daniel K. Lapsley. New York: Cambridge University Press; 2009:106–135.
- Nesse RM: **How can evolution and neuroscience help us understand moral capacities**? In *The Moral Brain: Essays on the Evolutionary and Neuroscientific Aspects of Morality.* Edited by Jan Verplaetse, Jelle De Schrijver, Sven Vanneste, Johan Braeckman. Netherlands: Springer; 2009 New York: Springer; 2009:201–209.
- Rose H: **Consciousness and the limits of neurobiology.** In *The New Brain Sciences: Perils and Prospects.* Edited by Dai Rees, Steven Rose. New York: Cambridge University Press; 2004:59–70.
- Rose S: **Having a brain, being a mind.** In his *The Future of the Brain: The Promise and Perils of Tomorrow's Neuroscience.* Oxford, U.K.: Oxford University Press; 2006:137–168.
- Roskies A: **What’s “neu” in neuroethics.** In *The Oxford Handbook of Philosophy and Neuroscience.* Edited by John Bickle. Oxford, U.K.: Oxford University Press; 2009:454–472.
- Roskies A: **A case study in neuroethics: the nature of moral judgment.** In *Neuroethics: Defining the Issues in Theory, Practice, and Policy.* Edited by Judy Illes. Oxford, U.K.: Oxford University Press; 2006:17–32.
- Schleim S: **Über einen möglichen normativen beitrag der Moralphysiologie.** In: *Brauchen wir eine neue Moral?: Herausforderungen der Ethik durch die Neurowissenschaft.* Edited by Gilbert Scharifi. Paderborn: Mentis; 2011:181–198.
- Suhler C, Churchland PS: **The neurobiological basis of morality.** In *The Oxford Handbook of Neuroethics.* Edited by Judy Illes, Barbara J. Sahakian. Oxford, U.K.: Oxford University Press; 2013:33–58.
- Tancredi L: **Neuroscience and morality.** In his *Hardwired Behavior: What Neuroscience Reveals about Morality.* New York: Cambridge University Press; 2005:1–11.
- Tancredi L: **Morality and the mind.** In his *Hardwired Behavior: What Neuroscience Reveals about Morality.* New York: Cambridge University Press; 2005:12–24.
- Tancredi L: **The moral brain.** In his *Hardwired Behavior: What Neuroscience Reveals about Morality.* New York: Cambridge University Press; 2005:34–45.
- Tancredi L: **Creating a moral brain.** In *Hardwired Behavior: What Neuroscience Reveals about Morality.* New York: Cambridge University Press; 2005:162–176.
- Tancredi LR: **The bad brain: biology of moral thinking.** In *The Variables of Moral Capacity.* Edited by David C. Thomasma and David N. Weisstub. Dordrecht: Kluwer; 2004:235–257.
- Zoloth L: **Being in the world: neuroscience and the ethical agent.** In *Neuroethics: Defining the Issues in Theory, Practice, and Policy.* Edited by Judy Illes. Oxford, U.K.: Oxford University Press; 2006:61–74.

## Discussion and conclusions

Despite our best efforts to amass as complete a bibliography of the past 10 years’ neuroethics literature as possible, automated indexing and other technical issues can affect the retrieval of documents. However, this need not constrain the capability of this document to provide a valuable nexus in, and for the discipline. As consistent with Part 1 of this series, we conceive of this bibliography as a participatory endeavor, and request that the readership contribute to this effort by adding any missing citations to the online comments section of this bibliography. Citations also can be emailed directly to the bibliographic manager at: **bioethics@georgetown.edu** for subsequent inclusion as commentary/addenda to this work.

To be sure, with advances in neuroscientific capabilities and expanding use of neuroscientific techniques and technologies in medicine, arguments are being made to address ethical issues generated by brain research [6], prompting elaboration of neuroethics as the “ethics of neuroscience” [2,7]. Indeed, it is (perhaps most) important to ask if such approaches to studying (moral) cognition and actions are technically apt, valid, and therefore of any real value [3,8]. Will necessary review, oversight and guidance be developed to direct and regulate if and how such research should or should not be conducted and translated into clinical treatments? Might studies of the putative neural bases of moral thought and action establish trends to engage these substrates and mechanisms in the clinical practices of neurology and psychiatry, and/or establish a basis for boutique, socially-, legally-, or politically-oriented interventions aimed at altering moral cognition and behaviors? Part 3 of this series will present a current bibliography of these and other neuroethical issues germane to clinical medicine.

In addition to implications for clinical care, neuroscientific studies of cognition, emotion and behavior can be – and are increasingly – leveraged in legal and social contexts, which must be considered on an international scale [9,10]. How, for example, might neuroscientific insights to the concept of free will incur consequences for questions of legal culpability? Can neuroscience provide metrics for, and standards of psychosocial “normality” and “abnormality” that are valid and viable within and across cultures? How will neuroscience and neurotechnologies be employed upon the twenty first century world stage to affect human health and capability, and evoke economic and political balances of power? Literature addressing these issues and questions will be presented in Part 4 of this bibliographic series. When taken together, we hope that this bibliography will elucidate the literature that is representative of the first ten years of this field, provide a historical view of this discipline’s growth, and afford insight to its developing canon.
